# Insular environment-dependent introgression from an arid-grassland orchid to a wetland orchid on an oceanic island

**DOI:** 10.1093/evlett/qrae034

**Published:** 2024-07-15

**Authors:** Kenji Suetsugu, Shun K Hirota, Masayuki Ishibashi, Kenya Ishida, Hiroshi Hayakawa, Yoshihisa Suyama

**Affiliations:** Department of Biology, Graduate School of Science, Kobe University, Kobe, Hyogo 657-8501, Japan; Institute for Advanced Research, Kobe University, 1-1 Rokkodai, Nada-ku, Kobe, Hyogo 657-8501, Japan; Field Science Center, Graduate School of Agricultural Science, Tohoku University, 232-3 Yomogida, Naruko-onsen, Osaki, Miyagi 989-6711, Japan; Botanical Gardens, Osaka Metropolitan University, 2000 Kisaichi, Katano City, Osaka 576-0004, Japan; Tokorozawa City, Saitama 359-0024, Japan; 184 Kozu Island village, Tokyo 100-0601, Japan; Museum of Natural and Environmental History, Shizuoka 5762 Oya, Suruga, 422-8017, Japan; Field Science Center, Graduate School of Agricultural Science, Tohoku University, 232-3 Yomogida, Naruko-onsen, Osaki, Miyagi 989-6711, Japan

**Keywords:** gene flow, hybridization, introgression, island biology, speciation

## Abstract

Adaptive introgression plays a vital role in allowing recipient species to adapt and colonize new environments. However, our understanding of such environment-dependent introgressions is primarily limited to specific plant taxa in particular settings. In Japan, two related orchid species, the autonomously self-pollinating *Pogonia minor* and the outcrossing *Pogonia japonica*, typically inhabit dry grasslands and wetlands, respectively. Intriguingly, an island ecotype of *P. japonica* exists in arid, wind-swept, open sites on volcanic mountain slopes on Kozu Island, in the oceanic Izu Islands. To investigate potential introgression and its implications between *P. japonica* and *P. minor* on Kozu Island, we applied a comprehensive approach that included examining morphological traits, genome-wide SNP data, and plastid DNA sequences. We also examined the breeding systems of these species on Kozu Island through artificial pollination experiments to determine if introgression from *P. minor* has endowed the *P. japonica* ecotype with selfing capabilities. Extensive sampling on Kozu Island revealed that all *P. japonica* specimens exhibit signs of introgression from *P. minor*, suggesting the absence of pure *P. japonica* populations on the island. Furthermore, the chloroplast haplotypes of the insular *P. japonica* ecotype consistently match those of *P. minor*, indicating a predominantly asymmetrical initial hybridization with *P. minor* acting mainly as the maternal parent in the formation of F1 hybrids. Despite the advantages of self-fertilization in isolated environments, the insular *P. japonica* does not exhibit autogamy. Consequently, the scarcity of moist habitats, rather than selection pressure for selfing, likely contributes to the observed widespread introgression. Our study strongly suggests that the arid-environment-adapted *P. minor* has introgressed into the insular ecotype of *P. japonica*, enabling its successful colonization of arid volcanic mountain slopes of the oceanic island.

## Introduction

Introgression is the process of transferring genetic material from one group of organisms to another through hybridization and recurrent backcrossing (Anderson, 1953; Goulet et al., 2017). Unlike other sources of genetic variation, such as standing variation and novel mutations, introgression can be advantageous because the genes that are transferred have already been adapted to the environment of the donor group (Anderson, 1948).

Consequently, although random processes may dominate in introgressive hybridization, adaptive introgression occurs when introgressed alleles are maintained by natural selection (Suarez-Gonzalez, Lexer & et al., 2018). Adaptive introgression has become recognized as a pivotal genetic reservoir for adaptation (Khodwekar & Gailing, 2017; Nagamitsu et al., 2020; Rieseberg et al., 2007; Suarez-Gonzalez et al., 2016; Suarez-Gonzalez, Hefer & et al., 2018). For example, *Helianthus annuus* ssp. *texanus*, a hybrid derived from *Helianthus debilis* and *H. annuus*, acquired enhanced resistance to herbivores from its *H. debilis* parent (Whitney et al., 2006). Similarly, the transfer of adaptive traits has been noted in the flood-resistant *Iris fulva* and the drought-tolerant *Iris brevicaulis* (Martin et al., 2006). In controlled backcrosses between these species, survival under severe flooding conditions was notably influenced by the introgressed alleles from *Iris fulva* in the genome (Martin et al., 2006).

While oceanic islands are invaluable for studying evolutionary processes and speciation (Baker, 1967; MacArthur & Wilson, 1967), introgression in oceanic islands remains underexplored, despite its potential significance. Although ancient oceanic islands often harbor endemic species with significant morphological divergence, complicating the elucidation of introgression events (Herben et al., 2005), younger oceanic islands may facilitate the corroboration of genetic dating of introgression events with the geological dating of the island age. Notably, Kozu Island, part of the Izu Islands, is known for its relatively recent emergence (ca. 0.3 Myr; Kaneoka et al., 1970). Volcanic eruptions, occurring from tens of thousands of years to as recently as a millennium ago, have also contributed to the youthfulness of the current vegetation (Sugihara et al., 2001).

Here, we focus on two related orchid species, *Pogonia minor* and *Pogonia japonica*, widely distributed in Japan. On the Japanese mainland, *P. japonica* typically inhabits oligotrophic wetlands, while *P. minor* is more commonly found in drier grassland habitats (Suetsugu, 2015b; Takahashi, 2015). However, *P. japonica* has been identified on Kozu Island (Ishibashi, 2011; Sugiyama, 1983; Tokyo Metropolitan Government, 2014). This insular ecotype of *P. japonica* has adapted to wind-swept, open sites on volcanic mountain slopes (Ishibashi, 2011). Due to the volcanic nature of the Izu Islands, moisture-rich habitats are scarce, resulting in a rarity of plant species adapted to wetlands (Inoue, 1993). Consequently, this environment has likely promoted the emergence of an ecotype suited to arid conditions.

Additionally, although most *P. japonica* individuals on Kozu Island (referred to as the insular ecotype of *P. japonica*) are apparently hardly distinguishable from their mainland counterparts, a population displaying intermediate morphological characteristics between *P. japonica* and *P. minor* (referred to as putative hybrids) has been discovered on the island (Ishibashi, 2011). This finding suggests that hybridization occurs at least in some individuals on Kozu Island. Based on this observation and the arid habitats of the insular ecotype of *P. japonica*, we hypothesize that widespread introgression from *P. minor* may be contributing to the niche adaptation of the insular ecotype.

It is also noteworthy that the two *Pogonia* species exhibit distinct pollination strategies: *P. japonica* has large flowers that are self-compatible but rely on pollinators for fruit set, while *P. minor* has smaller, self-pollinating flowers. The potential introgression of traits from *P. minor* to *P. japonica* could offer reproductive advantages by enhancing self-pollination, which is particularly useful in isolated populations (Barrett, 1996; Inoue et al., 1996), such as those on Kozu Island. In fact, in the Izu Islands, the number of pollinator species, particularly long-tongued ones, declines with increasing distance from the mainland (Fukasawa & Miyano, 2010; Hiraiwa & Ushimaru, 2017; Inoue, 1993). Thus, the potential introgression from *P. minor* may be adaptive, influencing not only habitat preferences but also shifts in the breeding system.

In this study, we aimed to explore the potential for introgression from *P. minor* to *P. japonica* on Kozu Island. We adopted a multifaceted approach, utilizing morphological characteristics, genome-wide SNP data, and plastid DNA (cpDNA) sequence data to investigate patterns of introgression between *P. japonica* and *P. minor*. Additionally, we examined the breeding systems of the insular ecotype of *P. japonica* and putative hybrids on Kozu Island to determine if introgression from *P. minor* has imparted selfing capabilities. Through these investigations, we assessed mutually non-exclusive hypotheses: introgressants may have been better suited to colonize this island either due to their autonomous selfing, providing reproductive assurance, or their drought tolerance, enhancing their establishment under arid conditions.

## Methods

### Study system

The Izu Islands, part of Tokyo Prefecture, Japan, serve as a valuable model for exploring evolutionary processes that contribute to island endemism (Nakahama et al., 2019; Suetsugu et al., 2024; Yamada & Maki, 2012; Yoichi et al., 2021). Unlike more isolated oceanic islands, these islands are located relatively close to the Japanese mainland, Honshu. The chain stretches from north to south, 25 to 250 kilometers off the Honshu coast. Despite this proximity, the Izu Islands maintain a moderate degree of isolation and host thirty-nine endemic vascular plant taxa, likely evolved from mainland ancestral species (Nakahama et al., 2019; Ohba & Akiyama, 2002).

**Figure 1.**
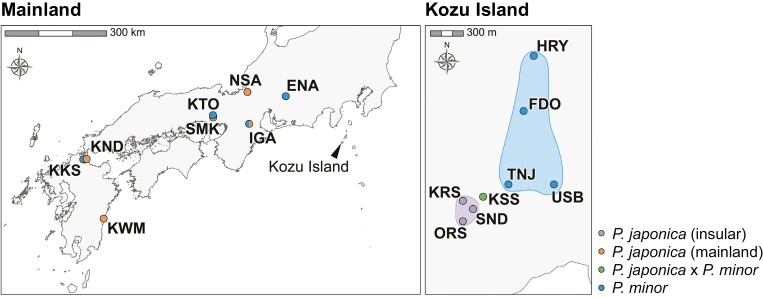
Map showing the sampling localities of mainland *Pogonia japonica*, the insular ecotype of *P. japonica*, *P. japonica* × *P. minor*, and mainland and insular *P. minor*. Areas outlined in light color represent the habitats of insular *P. japonica* and *P. japonica* × *P. minor* on Kozu Island.

Furthermore, the Izu Islands are known for their recent geological history. Kozu Island, a gourd-shaped volcanic island measuring approximately 4 km east to west and 6 km north to south, formed about 0.3 Myr ago (Kaneoka et al., 1970; Kitazato, 1997). This relatively young geological status is amplified by a sequence of significant volcanic events spanning tens of thousands of years, culminating in the most recent eruption roughly a thousand years ago (Sugihara et al., 2001; Takahashi. et al., 2022). Therefore, Kozu Island, with its recent geological formation and close proximity to the mainland—approximately 50 km at its nearest point—presents an ideal model for investigating the evolutionary dynamics between oceanic islands and mainland regions (Figure 1; Inoue, 1993; Suetsugu et al., 2024).

### Study species and sampling scheme

*Pogonia japonica* is pollinated by the bee species *Ceratina flavipes* (Hymenoptera, Xylocopinae; Ushimaru & Nakata, 2001), while no pollinators have been documented for *P. minor*. Although *P. minor* closely resembles *P. japonica*, it can be distinguished by its smaller flowers and a more closed perianth tube, due to its primarily autonomous self-pollination mode (Suetsugu, 2015b).

Reciprocal F1 hybrids between these species show high embryo formation (approximately 90%) and germination rates (over 80%; Takahashi, 2015). The F2 reciprocal hybrids have embryo formation rates around 80%, but lower germination rates compared to the parental species and F1 hybrids, specifically 26.0% in *P. japonica* (female) × *P. minor* (male) F1 hybrids and 4.2% in *P. minor* (female) × *P. japonica* (male) F1 hybrids. Despite these low germination rates, F2 hybrids from reciprocal crosses exhibit vigorous seedling growth post-germination (Takahashi, 2015). These findings suggest the potential for natural hybrid formation that might propagate both vegetatively and through seed production.

**Figure 2.**
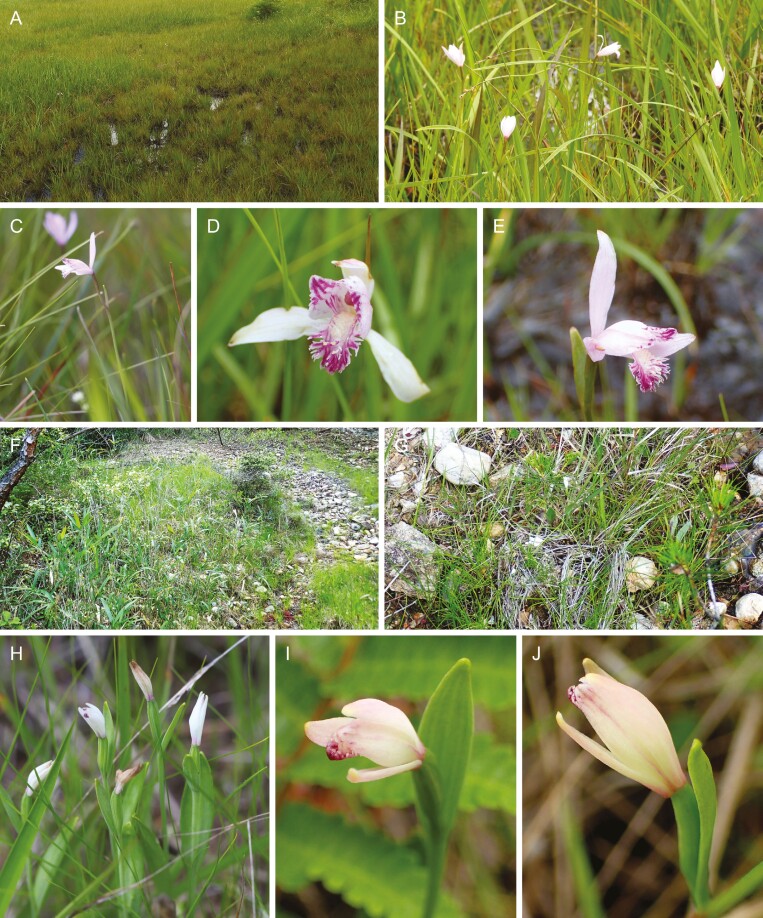
The contrasting habitats of *Pogonia japonica* and *P. minor* on mainland Japan (Ena City, Gifu Prefecture). (A, B) The wetland harboring flowering plants of *P. japonica.* (C–E) Flowering plants of *P. japonica* in their wetland habitats. (F–G) The dry grassland harboring flowering plants of *P. minor*. (H–J) Flowering plants of *P. minor* in their dry grassland habitats.

**Figure 3.**
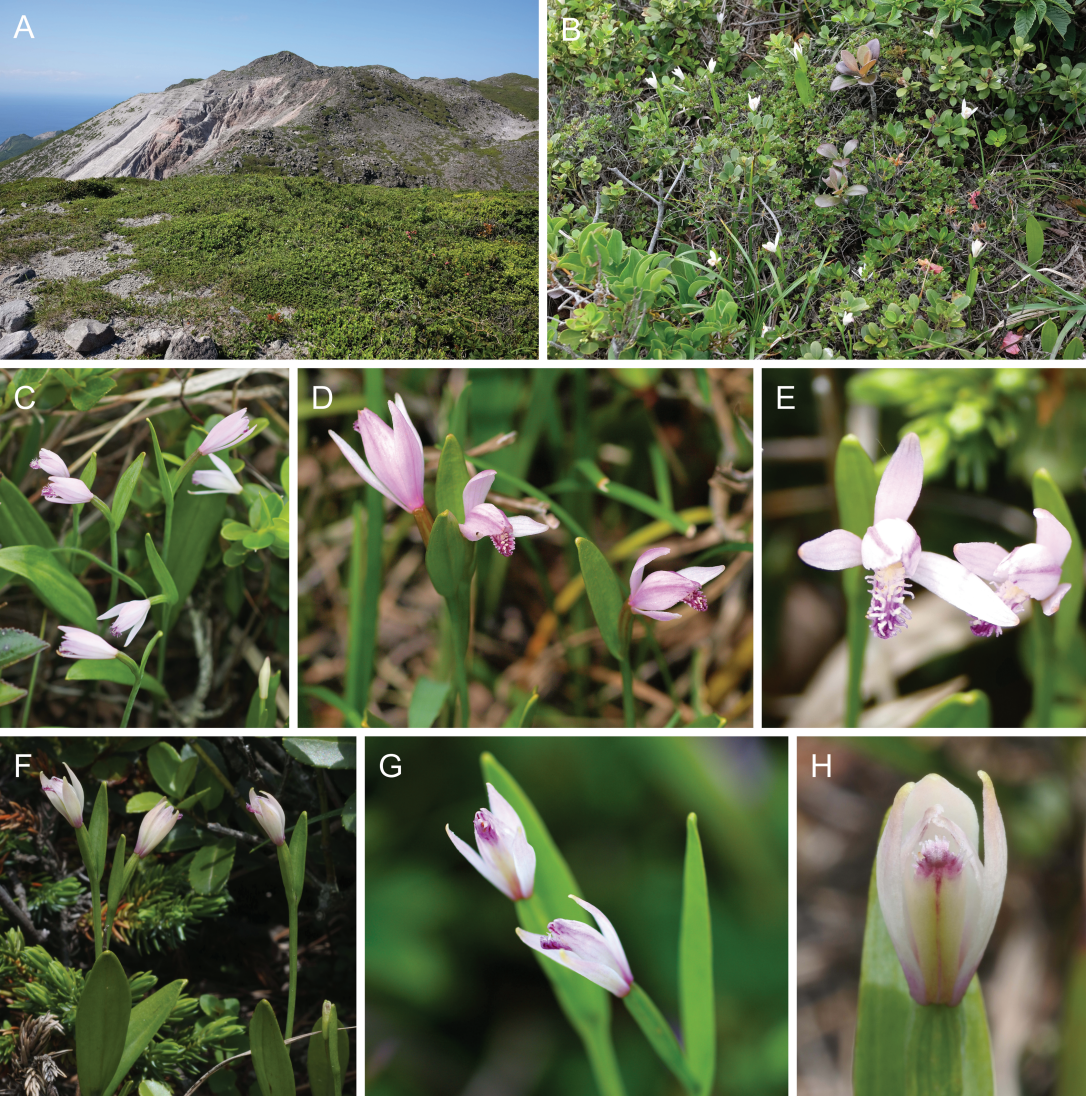
The habitats and flowering plants of the insular ecotype of *Pogonia japonica* and *P. minor* on Kozu Island. (A, B) The dry and wind-swept site with flowering *P. japonica* plants. (C–E) Flowering *P. japonica* plants growing among shrubs in their dry and wind-swept habitats. (F–H) Flowering *P. minor* plants growing among shrubs in their dry and wind-swept habitats.

**Figure 4.**
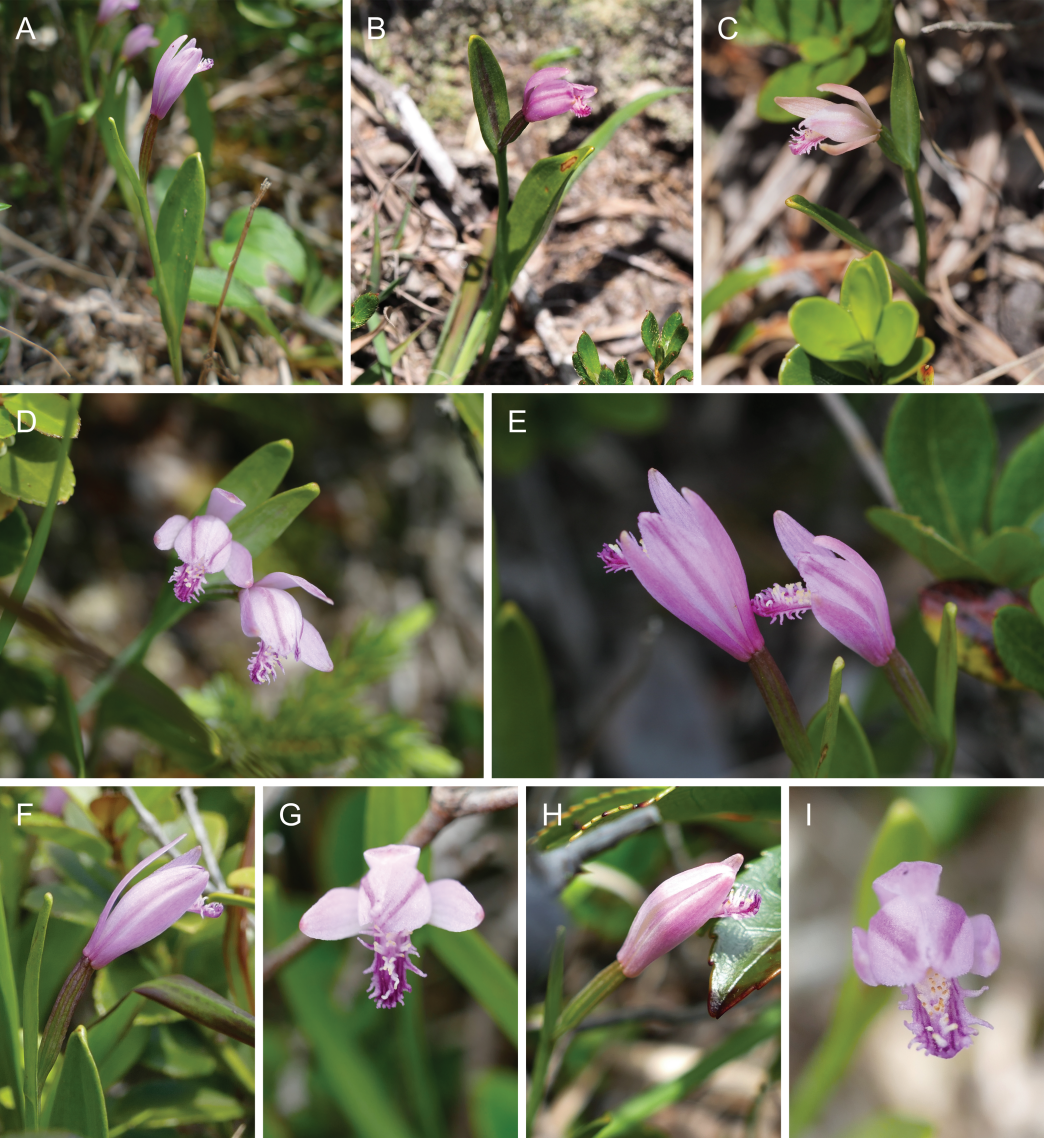
The habitats and flowering plants of *Pogonia japonica* × *P. minor* on Kozu Island. (A–E) Flowering plants growing among shrubs in their dry and wind-swept habitats. (F–I) Close-up of flowers.

Despite these potential interactions, natural interspecific hybridization between these two species has not yet been observed in their natural habitats (Takahashi, 2015). Contributing factors to this absence may include the nearly obligate selfing nature of *P. minor* (Suetsugu, 2015b) and divergent habitat preferences—wetlands for *P. japonica* and arid grassland for *P. minor* (Figure 2, Supplementary Figure S1). However, on Kozu Island, both species are found in similar environments (Figure 3), such as mountain slopes and ridges with abundant sunlight and well-drained soil (Ishibashi, 2011), increasing the likelihood of hybrid formation. Indeed, putative hybrids have been noted on this island, distinguishable from *P. japonica* by their smaller dorsal sepal length (approximately 15 mm vs. 20 mm) and a less open perianth tube (Figure 4), while the insular ecotype of *P. japonica* is nearly indistinguishable from the mainland *P. japonica* (Supplementary Figures S2 and S3).

Long-term monitoring conducted by Masayuki Ishibashi and Kenya Ishida has identified approximately 200 individuals of the insular ecotype of *P. japonica* across a 250 m × 200 m area on Kozu Island. Although these individuals occupy a relatively confined space, which might suggest a single population, they are distributed into smaller clusters. To better understand potential genetic differences, such as the degree of genetic mixing, we have divided these insular *P. japonica* individuals into three localities. Each locality is located at least 200 meters apart: Kuroshima (KRS), Orosha (ORS), and Seidai-ike (SND). Additionally, about 60 putative hybrid individuals are concentrated within a much smaller area of 20 m × 20 m and are treated as one group, with the population name Kuroshima-shita (KSS; Figure 1).

For survey sites on the mainland, *P. japonica* and *P. minor* samples collected within 1 km of each other were considered to be from the same location. However, on Kozu Island, to more precisely assess the effects of location on genetic characteristics, both *P. japonica* and *P. minor* samples collected more than 200 m apart were categorized as originating from different localities (Supplementary Tables S1 and S2). Meteorological, soil type, and rock type data for sampling sites were obtained from https://nlftp.mlit.go.jp/ksj/gml/datalist/KsjTmplt-G02-2022.html, https://gbank.gsj.jp/seamless/use.html and https://nlftp.mlit.go.jp/kokjo/inspect/landclassification/download.html, respectively.

### Morphological observations

We conducted a morphological analysis involving 40 individuals of *P. japonica* (17 from 5 mainland sites and 23 from three sites on Kozu Island), 26 individuals of *P. minor* (13 from 5 mainland sites and 13 insular individuals from three sites on Kozu Island), and 16 putative hybrid individuals from the KSS population on Kozu Island (Supplementary Table S1). For the insular ecotype of *P. japonica* and the putative hybrids, the aforementioned sampling sites encompass all the currently known localities (Figure 1). Additionally, we ensured that the sampling was as evenly distributed as possible across each site to capture a representative range of morphological variability within these populations.

To quantify the morphological variations, we measured lengths and widths of the following floral structures using a digital caliper: ovary, dorsal sepal, lateral petal, lateral sepal, labellum, and column. To assess differences among mainland *P. japonica*, insular *P. japonica*, mainland *P. minor*, insular *P. minor*, and putative hybrids, we performed one-way ANOVA tests for each trait. Where significant differences were observed, post-hoc multiple comparisons were made using the Tukey–Kramer test.

To summarize the overall patterns of floral variation, we used a permutational multivariate analysis of variance (PERMANOVA) with 9999 permutations using the adonis2 function from the vegan package. Post hoc comparisons for all PERMANOVAs were conducted using Bonferroni-corrected pairwise PERMANOVAs, utilizing the pairwise.adonis function from the pairwiseAdonis package. Principal component analysis (PCA) was also generated to visualize the differences in floral traits among groups and assist in interpreting the PERMANOVA results. All statistical analyses were performed using R software, version 3.6.0.

### Breeding systems

Considering previous studies suggest that autogamy has evolved in plants that are bee-pollinated on the mainland (Inoue, 1993; Inoue & Amano, 1986; Yamada & Maki, 2014), we investigated the reproductive strategies of the insular ecotype of *P. japonica* in Kuroshima, Kozu Island, and the putative hybrids in Kuroshima-shita, Kozu Island.

We executed hand-pollination experiments with four distinct treatments: (i) autonomous autogamous treatment—flowers were shielded in fine-mesh nets before anthesis to block pollinator access (10 flowers from 10 individuals); (ii) artificial self-pollination treatment—pollinaria were manually extracted and used to pollinate the same flower, which was subsequently enclosed in a fine-mesh net (10 flowers from 10 individuals); (iii) artificial cross-pollination treatment—similar to artificial self-pollination, but pollinaria were obtained from a different plant (10 flowers from 10 individuals); (iv) open treatment—flowering plants were randomly marked and allowed to fruit under natural conditions (50 flowers from 50 individuals in the insular ecotype of *P. japonica* and 10 flowers from 10 individuals in the putative hybrids). We monitored fruit set over 6 to 8 weeks and compared the results statistically using Fisher’s exact test.

Given that autonomous self-pollination in orchids is often enabled by alterations in column morphology (Suetsugu, 2015a, 2015b), and artificial pollination experiments indicate that this self-pollination is absent in the insular ecotype of *P. japonica* but present in the putative hybrids (see *Results*), we investigated the floral structures of five plants from each group. These plants were previously isolated in nylon mesh bags to exclude pollinators and were examined approximately three days post-flower opening.

### MIG-seq- and MPM-seq-based high-throughput genomic library processing

Genomic data for *P. japonica* and *P. minor* were acquired through a complementary application of two high-throughput DNA sequencing technologies: MIG-seq and MPM-seq (Suyama et al., 2022). MIG-seq, a genome-wide genotyping technique, exclusively sequences the ISSR region, enabling efficient SNP detection even in species with large genome sizes, such as *Pogonia* species (Leitch et al., 2009; Suyama et al., 2022). Conversely, MPM-seq targets multiple barcoding regions, focusing on maternally inherited chloroplast DNA. This combined approach provides robust evidence, particularly for identifying putative hybrids that often require confirmation via multiple independent phylogenetic markers (Suyama et al., 2022). This approach is also valuable for analyzing interspecies gene flow, revealing insights into both the parental species and their maternal lineages (Suyama et al., 2022).

We collected samples from 50 *P. japonica* individuals, including 27 from the insular ecotype from all currently recognized localities (KRS, ORS, and SND), 42 *P. minor* individuals across various Japanese localities, and 7 putative hybrid individuals from the KSS population on Kozu Island (Supplementary Table S2). We ensured that the sampling was as evenly distributed as possible across each site to capture a representative range of genetic variability within these populations. Genomic DNA was extracted from their silica-dried leaves using the cetyltrimethylammonium bromide method (Doyle & Doyle, 1990). We prepared MIG-seq libraries for all samples and MPM-seq libraries for 19 *P. japonica* individuals (including 13 from the insular ecotype of the three localities), 11 *P. minor* individuals, and 5 putative hybrids, according to protocols by Suyama et al. (2022). These libraries were sequenced on an Illumina MiSeq Sequencer using a MiSeq Reagent Kit v3 (150 cycles) for MIG-seq and a MiSeq Reagent Nano Kit v2 (500 cycles) for MPM-seq. We deposited the MIG-seq and MPM-seq raw reads in the DDBJ Sequence Read Archive (BioProject Accessions: MIG-seq data—PRJDB17977; MPM-seq data—PRJDB17978).

For MIG-seq, we obtained a total of 13,743,346 reads (with an average of 138,822 ± 3,750 reads per sample) from the initial 15,597,072 raw reads (with an average of 157,546 ± 4,291 per sample) following the removal of primer sequences and low-quality reads (Suetsugu et al., 2021). For de novo SNP discovery, the Stacks 2.65 pipeline was used (Rochette et al., 2019), with the following parameters: a minimum depth of coverage to create a stack (*m*) of 3, a maximum distance allowed between stacks (*M*) of 2, and the number of mismatches allowed between sample loci while building the catalog (*n*) of 2. Using the ‘population’ program in Stacks, SNP sites with high heterozygosity (Ho ≥ 0.6) were removed, and SNP sites with fewer than three minor alleles were filtered out. To prevent the inclusion of linked SNPs, only the first SNP from each locus was considered. Depending on the specific objectives of our various analyses, we utilized four distinct SNP datasets.

For MPM-seq, we sequenced two chloroplast genomic regions—*trnL* intron and *rbcL*—along with the nuclear internal transcribed spacer (ITS1). The output comprised 33,656 reads for ITS1 (961 ± 73 reads per sample), 71,022 reads for rbcL (2,029 ± 118 reads per sample), and 20,912 reads for the trnL intron (597 ± 47 reads per sample). We analyzed the sequences using the Claident pipeline version 0.2.2019.5.10 (Tanabe & Toju, 2013), aligning the sequence data initially with MAFFT version (Katoh et al., 2009) and manually adjusting for optimal alignment. For ITS1, paired-end reads were merged where overlapping, whereas for *rbcL* and the *trnL* intron, paired-end reads were processed separately due to their short lengths.

### Phylogenetic, population structure, and gene flow analysis

To elucidate the origins of the insular ecotype of *P. japonica* and the putative hybrids, we initially used the following analytical approaches: SNP-based maximum likelihood phylogeny, Neighbor-Net network analysis, and STRUCTURE analysis. SNPs were filtered with a minimum proportion threshold of 0.7 for samples retaining each SNP (‘populations’ parameter *R* = 0.7), resulting in 946 SNPs from 99 samples for further analyses. We reconstructed the maximum likelihood phylogeny using RAxML 8.2.10 (Stamatakis, 2014), with a GTR substitution model and Lewis” ascertainment bias correction, involving 1,000 bootstrapping iterations. The Neighbor-Net network was constructed using SplitsTree4 4.14 (Huson & Bryant, 2006), based on the uncorrelated *p* distance matrix, ignoring ambiguous sites. Population structure was examined using STRUCTURE 2.3.4 (Pritchard et al., 2000), with 30 independent runs, a burn-in of 100,000 steps followed by 100,000 MCMC steps, and log-likelihoods estimated for each cluster (*K* = 1–10). Optimal *K* values were determined using the Delta *K* method (Evanno et al., 2005) in Structure Harvester (Earl & vonHoldt, 2012), with clustering results visualized via CLUMPAK (Kopelman et al., 2015). Population statistics, including the number of private alleles, observed heterozygosity (*H*_O_), expected heterozygosity (*H*_E_), the average inbreeding coefficient (*F*_IS_), and nucleotide diversity (*π*), were also calculated using the ‘populationʼ program within the Stacks software.

Given that the aforementioned initial genetic analyses identified (i) the insular ecotype of *P. japonica* as an introgressant and (ii) the putative hybrids as actual hybrids, we used the ‘introgressʼ package (Gompert & Buerkle, 2009, 2010) in *R* to assess their genetic admixture, calculating a hybrid index and interspecific heterozygosity. The hybrid index quantifies the proportion of alleles from one parental species, while interspecific heterozygosity measures heterozygosity for alleles from both parents, akin to the methodology in NewHybrids (Anderson & Thompson, 2002), but with fewer assumptions about linkage and selection (Milne & Abbott, 2008; Walsh et al., 2015).

For the ‘introgress’ analysis, samples were grouped based on insights from the Neighbor-Net and STRUCTURE analyses: mainland *P. japonica*, insular ecotype of *P. japonica*, hybrids, and mainland and insular *P. minor*. SNPs common to 50% or more of the samples in each group were selected (‘populations’ parameter at *r* = 0.5 and *p* = 3), with a minor allele frequency cutoff of 5%. Ultimately, we selected 699 SNPs for the ‘introgressʼ analysis. The hybrid index varied from 0 (pure *P. japonica*) to 1 (pure *P. minor*), with interspecific heterozygosity ranging from 0 (all homozygous) to 1 (all heterozygous). Furthermore, to examine the hypothesis that the hybrid population originated by hybridization of insular *P. japonica* and *P. minor*, the ‘introgress’ analysis was also performed for insular *P. japonica*, *P. minor*, and the hybrids. SNPs common to 50% or more of the samples in each group were selected (‘populations’ parameter at *r* = 0.5 and *p* = 3), with a minor allele frequency cutoff of 5%. Ultimately, we selected 787 SNPs. In this sample set, the hybrid index varied from 0 (insular *P. japonica*) to 1 (pure *P. minor*). F1 hybrids were expected to have a hybrid index of 0.5 and heterozygosity of 1, while F2 hybrids and backcrosses typically show reduced heterozygosity. Individuals with intermediate hybrid index values (0.25 to 0.75) and high heterozygosity (> 0.3) were classified as recent-generation hybrids (F1, F2), following Milne & Abbott, (2008).

Finally, to estimate the initial lineage split and population admixture events among the groups, we utilized the Approximate Bayesian Computation (ABC) algorithm in DIYABC software version 2.1.0 (Cornuet et al., 2014), applying the same SNP dataset from the ‘introgress’ analysis. We examined seven scenarios (Supplementary Table S4, Figure S4), based on the Neighbor-Net network and STRUCTURE analyses. Scenarios 1 and 2 propose that the insular ecotype of *P. japonica* and the hybrid population (KSS) originated from two independent admixture events. Scenario 3 suggests that both the insular population of *P. japonica* and the hybrid population (KSS) emerged from an admixed population of 2 species. Scenarios 4 and 5 hypothesize that the hybrid population (KSS) arose from an admixture event involving the insular population of *P. japonica* and either *P. minor* or *P. japonica.* Scenarios 6 and 7 postulate that the insular population of *P. japonica* developed from an admixture event involving the hybrid population (KSS) and either *P. minor* or *P. japonica.* We integrated 16 types of summary statistics (Supplementary Table S5) as outlined by DIYABC and executed 1,000,000 simulations for each scenario to identify the most probable scenario by comparing the posterior probabilities.

## Results

### Meteorological and soil type data

All sites where the insular ecotype of *P. japonica* is found are characterized by volcanic rocks formed in the post-Holocene era (less than 11,700 years ago; Supplementary Table S3). These volcanic rocks are known for their well-draining properties (Tsuya, 1930), which makes them particularly prone to drying out. Conversely, Kozu Island, compared to other habitats of *Pogonia*, receives a higher amount of precipitation (Supplementary Table S3), potentially allowing the insular *P. japonica* to survive despite these challenging conditions.

### Morphological observations

Floral morphological traits exhibited significant disparities between *P. japonica* and *P. minor*, with the former generally displaying larger floral components (PERMANOVA: *F*-value = 66.8, *R*^2^ = 0.63, *P* < 0.001; Supplementary Table S6, Figures S5–S12). Notably, some floral parts in the insular ecotype of *P. japonica* (later genetically identified as an introgressant; see the *Molecular Section*) were smaller than those in mainland populations (Supplementary Figure S13). Additionally, nearly all the morphological characteristics of the putative hybrids (later genetically confirmed as actual hybrids; see the *Molecular Section*) displayed significant reductions compared to those of *P. japonica* (Supplementary Figures S10 and S11). In the putative hybrids, the lengths and widths of almost all floral parts were intermediate between those of *P. japonica* and *P. minor* (Supplementary Figure S13). Conversely, there were no discernible differences in the floral morphological traits of *P. minor* between the insular and mainland populations (Supplementary Figures S7, S12, S13).

**Figure 5.**
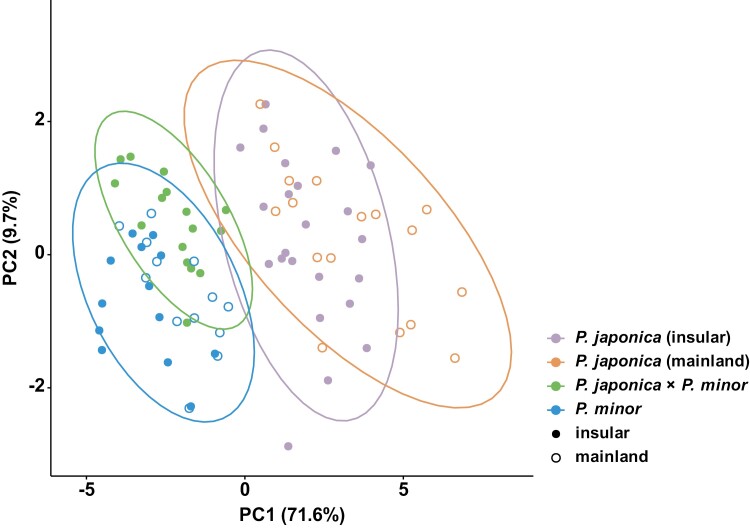
Results of the principal component analysis, revealing morphological characteristics of mainland *Pogonia japonica*, the insular ecotype of *P. japonica*, *P. japonica* × *P. minor*, and mainland and insular *P. minor*, based on the following floral traits: Lo (length of ovary), Ld (length of dorsal sepal), Llp (length of lateral petal), Lls (length of lateral sepal), Ll (length of labellum), Lc (length of column), Wo (width of ovary), Wd (width of dorsal sepal), Wlp (width of lateral petal), Wls (width of lateral sepal), Wl (width of labellum), and Wc (width of column).

The PERMANOVA and PCA results, incorporating all floral traits, showed marginally significant differentiation between the insular and mainland populations of *P. japonica* (Bonferroni-corrected pairwise PERMANOVAs: *F*-value = 5.9, *R*^2^ = 0.13, *P* = 0.051; Supplementary Table S7). However, such differentiation was not observed between the insular and mainland populations of *P. minor* (Bonferroni-corrected pairwise PERMANOVAs: *F*-value = 3.0, *R*^2^ = 0.11, *P* = 0.494; Supplementary Table S7). It is noteworthy that the differences observed between the insular *P. japonica* and mainland *P. japonica* were substantially less pronounced than those between hybrids of *P. japonica* and *P. minor*, and the mainland *P. japonica* (Bonferroni-corrected pairwise PERMANOVAs: *F*-value = 71.8, *R*^2^ = 0.70, *P* < 0.001; Figure 5).

### Breeding system

Pollination experiments revealed distinct reproductive strategies between the insular ecotype of *P. japonica* and hybrids between *P. japonica* and *P. minor*. The insular ecotype of *P. japonica* is entirely reliant on pollinators for successful fruit set. Our results indicate a fruit set of 100% for artificially self-pollinated flowers and 90% for cross-pollinated flowers. However, flowers from which pollinators were excluded showed a fruit set of 0%. Among open-pollinated flowers, a significantly lower fruit set of 26% was observed, highlighting pollinator dependence (Fisher’s exact test: Cramer’s *V* = 0.57, *P* < 0.001 in open-pollination flowers vs. self-pollinated flowers and Cramer’s *V* = 0.50, *P* < 0.001 in open-pollination flowers vs. cross-pollinated flowers). Morphological investigation into the column revealed that the presence of a rostellum effectively separates the stigma and pollinia, making autogamy unlikely in not only mainland but also insular *P. japonica* (Supplementary Figures S5, S6, S8, S9).

In contrast, hybrids between *P. japonica* and *P. minor* displayed no significant variations in fruit set across different pollination treatments: 90% for artificial self-pollination, 100% for artificial cross-pollination, 70% for pollinator-excluded conditions, and 80% for open pollination (Fisher’s exact test: Cramer’s *V* = 0.14, *P* = 0.50 in open-pollination flowers vs. self-pollinated flowers, Cramer’s *V *= 0.33, *P* = 0.24 in open-pollination flowers vs. cross-pollinated flowers, and Cramer’s *V* = 0.12, *P* = 0.50 in open-pollination flowers vs. pollinator-excluded flowers). Column morphology investigations revealed a significant reduction or absence of the rostellum in not only *P. minor* but also hybrids between *P. japonica* and *P. minor* (Supplementary Figure S7, S10–S12). This degenerated rostellum in these hybrid individuals allows for direct contact between the pollinia and stigma, facilitating autonomous self-pollination. Additionally, we found that stigma exudates occasionally aid in the dispersion of pollen grains across the stigma lobe in these hybrids. This suggests that these hybrids are autonomously self-pollinating and not constrained by the availability of pollinators under natural conditions.

### Phylogenetic, population structure, and gene flow analysis

**Figure 6.**
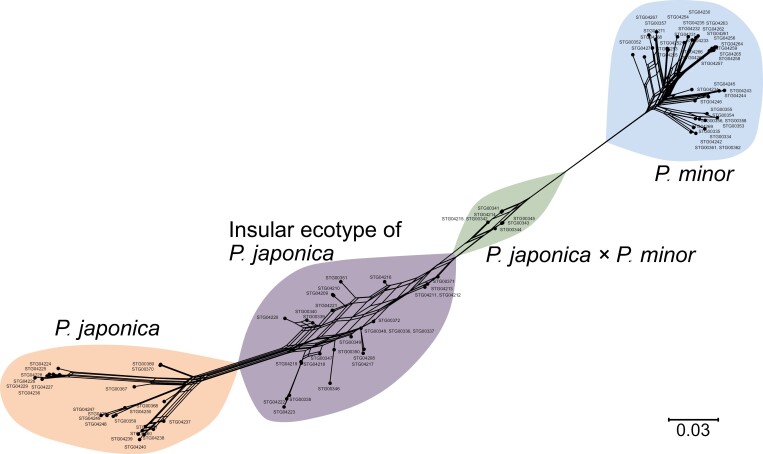
Neighbor-Net network of mainland *Pogonia japonica*, the insular ecotype of *P. japonica*, *P. japonica* × *P. minor*, and mainland and insular *P. minor* reconstructed based on the uncorrected *p* distance.

**Figure 7.**
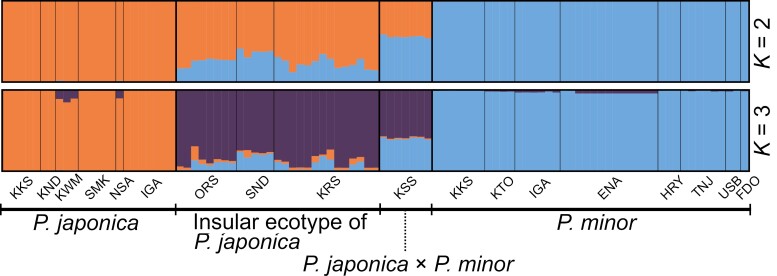
Population structure of mainland *Pogonia japonica*, the insular ecotype of *P. japonica*, *P. japonica* × *P. minor*, and mainland and insular *P. minor*, inferred with STRUCTURE 2.3.4. Taxa and populations are separated by broad and narrow vertical black lines, respectively.

MIG-seq-based high-throughput genomic data provided evidence that putative hybrids on Kozu Island are actual hybrids. The maximum likelihood and Neighbor-Net phylogenetic analyses indicated that they occupy a phylogenetic space intermediate to *P. japonica* and *P. minor* (Figure 6, Supplementary Figure S14). Additionally, the STRUCTURE analysis demonstrated that these hybrids contain genetic components from both parent species. Moreover, both phylogenetic and STRUCTURE analyses indicated that the insular ecotype of *P. japonica* also contains some genetic components of *P. minor*, suggesting that they are introgressants. Notably, the STRUCTURE analysis indicated Delta *K* was highest at *K *= 2 (Supplementary Figure S15). At *K *= 2, mainland *P. japonica* and *P. minor* were separated into distinct genetic clusters, while both hybrids and the insular ecotype of *P. japonica* exhibited a mixture of both genetic clusters. This is consistent with the observations that insular *P. japonica* and hybrids showed higher nucleotide diversity than mainland *P. japonica* and *P. minor*, possibly due to possessing unique alleles from both *P. japonica* and *P. minor* (Supplementary Table S8). At *K *= 3, it revealed three distinct groups: (i) mainland *P. japonica*, (ii) mainland and insular *P. minor*, and (iii) insular *P. japonica* (Figure 7). The STRUCTURE analysis at *K* = 3 also suggested that the hybrids likely originated from hybridization events between the already introgressed insular *P. japonica* and pure *P. minor*.

**Figure 8.**
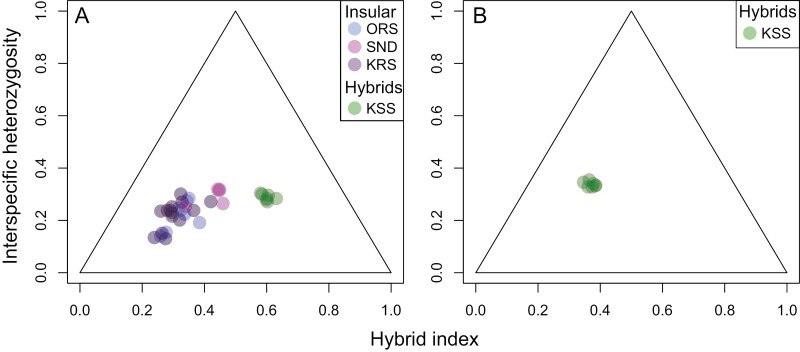
Triangle plot of interspecific heterozygosity and hybrid index for the insular ecotype of *Pogonia japonica* (locations: Orosha [ORS], Sendai-ike [SND], and Kuroshima [KRS]) and the hybrid *P. japonica* × *P. minor* (location: Kuroshima-shita [KSS]) on Kozu Island, the Izu Islands. The hybrid index is measured as the proportion of alleles with *P. minor* ancestry, ranging from 0 (indicating exclusively *P. japonica*-derived alleles) to 1 (indicating exclusively *P. minor*-derived alleles: A) and 0 (indicating exclusively the insular ecotype of *P. japonica*-derived alleles) to 1 (indicating exclusively *P. minor*-derived alleles: B).

MPM-seq analysis underscores consistent genetic differences between *P. japonica* and both mainland and insular *P. minor*, particularly in the mononucleotide repeat polymorphisms in the *trnL* intron of cpDNA and two polymorphic sites in the ITS1 region. However, *rbcL* sequences are identical between *P. japonica* and *P. minor*. In the ITS1 region, all individuals of the insular ecotype of *P. japonica* and the hybrids share the same genotype as mainland *P. japonica*. Among the six examined mainland *P. japonica* individuals, two exhibited 19 bp repeats while four displayed 18 bp in the *trnL* intron. In contrast, all 11 *P. minor* individuals possessed 16 bp repeats in this region, a pattern also seen in all five hybrid individuals. Among the 13 insular ecotype individuals of *P. japonica*, one had 19 bp of repeats, ten had 16 bp, and two had 15 bp in the *trnL* intron. This pattern indicates that the majority of both the hybrids (5/5) and the insular ecotype (12/13) possess the *P. minor* cpDNA haplotype, suggesting an asymmetrical initial hybridization event with *P. minor* predominantly acting as the maternal parent during the formation of the F1 hybrids. Consistent with our findings from phylogenetic, population structure, and cpDNA analyses, the ‘introgress’ package confirms that the insular ecotype of *P. japonica* contains genetic components from *P. minor.* However, the extent of this genetic contribution from *P. minor* differs between hybrid individuals and insular *P. japonica* individuals. All the hybrid individuals exhibited over 50% nuclear germplasm identical to *P. minor*, with a hybrid index of 0.601 ± 0.006, ranging from 0.581 to 0.631. Conversely, all individuals of the insular ecotype of *P. japonica* showed over 50% nuclear germplasm identical to *P. japonica*, with a hybrid index range of 0.333 ± 0.012, ranging from 0.239 to 0.460. Individuals of the insular *P. japonica* showed interspecific heterozygosities ranging from 0.130 to 0.319 (average: 0.234 ± 0.011), suggesting that most insular *P. japonica* individuals are genetically admixed hybrids of later generations. Furthermore, the insular ecotype of *P. japonica* possessed 65 private alleles, and its observed heterozygosity (*H*_*O*_) was close to the expected heterozygosity (*H*_*E*_), while the hybrid individuals had only one private allele and exhibited a higher *H*_*O*_ (Supplementary Table S8), indicating that the insular ecotype likely originated from an earlier admixture event. Although no clear genetic differences were observed among the insular *P. japonica* individuals collected from different locations, individuals sampled from the Sendai-ike population exhibited slightly higher tendencies in both hybrid index and interspecific heterozygosity (Figure 8A).

All individuals of the hybrids showed heterozygosity close to 0.3, ranging from 0.271 to 0.304 (0.288 ± 0.004). In the ‘introgress’ analysis using insular *P. japonica*, *P. minor*, and the hybrids, the hybrid population showed higher interspecific heterozygosity (0.338 ± 0.003, ranging from 0.328 to 0.355) (Figure 8B). At the population level, the hybrid population showed higher *H*_*O*_ than *H*_*E*_ and the lowest *F*_IS._ In contrast, *P. minor*, which has a primarily autonomous self-pollination mode similar to the hybrids, exhibited lower *H*_*E*_ than *H*_*O*_ and a relatively higher population-level *F*_IS_ than the bee-pollinated *P. japonica* (Supplementary Table S8). These results suggest that the hybrid population consists of recent-generation hybrids.

**Figure 9.**
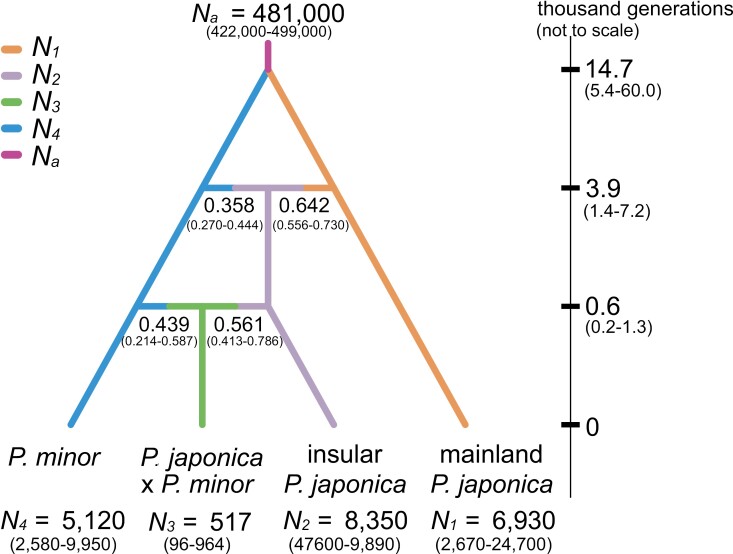
Demographic history of *Pogonia japonica* and *P. minor* estimated by DIYABC. Effective population sizes (*N*), times since divergence and admixture events (in thousand generations, not to scale), and admixture proportions were derived from the median and 95% CI of posterior parameter distributions (Supplementary Table S9).

The demographic history was further supported by ABC methods. Among the seven scenarios, Scenario 4 had the highest posterior probability (direct approach: 0.4140, 95% CI 0.0000–0.8457; logistic approach: 0.9374, 95% CI 0.9263–0.9485). This finding suggests that the hybrid population (KSS) descended from an admixture event between the insular population of *P. japonica* and *P. minor*. The scenarios with the second-highest posterior probabilities were Scenario 2 (direct approach: 0.2300, 95% CI 0.0000–0.5989; logistic approach: 0.009, 95% CI 0.0003–0.0016) and Scenario 7 (direct approach: 0.0660, 95% CI 0.0000–0.2836; logistic approach: 0.0561, 95% CI 0.0451–0.0670). Scenario 4 estimated the median divergence time between *P. japonica* and *P. minor* at 14,700 generations ago (5,390–60,000), and the insular ecotype of *P. japonica* is estimated to have arisen from an admixture event between *P. japonica* and *P. minor* about 3,850 generations ago (1,400–7,170; Fig. 9). The hybrid population (KSS) is believed to have descended from an admixture event between the insular ecotype of *P. japonica* and *P. minor* approximately 690 generations ago (158–1,290; Supplementary Table S9).

## Discussion

We have shown that not only the putative hybrids but also the insular ecotype of *P. japonica*, exhibit genetic admixture with *P. minor*. This study provides the first evidence of interspecific hybridization between these two species in their natural habitats. Comprehensive sampling revealed that all specimens of the insular *P. japonica* show signs of introgression with *P. minor*. Consequently, it is highly probable that pure *P. japonica* may no longer exist on Kozu Island. Our molecular analyses, including STRUCTURE and ABC analyses, have indicated that the hybrids on Kozu Island result from hybridization not between pure *P. japonica* and *P. minor*, but rather between insular *P. japonica* (already introgressed with *P. minor*) and pure *P. minor*. Moreover, given that the nuclear genetic composition of insular *P. japonica* leans more towards that of pure *P. japonica*, our results strongly suggest a more frequent occurrence of backcrossing with pure *P. japonica*—which, although now extinct on Kozu Island, likely existed there previously—after the genesis of F1 hybrids.

The insular ecotype of *P. japonica* has historically been considered “pure” due to its morphological similarity to the mainland population of *P. japonica* (Ishibashi, 2011; Sugiyama, 1983; Tokyo Metropolitan Government, 2014). Our data partially support this view, as the nuclear genome of the insular population is predominantly derived from *P. japonica*, and detailed morphological analysis confirms its close resemblance to its mainland counterpart. However, it is also important to note that the insular *P. japonica* contains genetic elements from *P. minor*, suggesting that its ecotype formation is primarily influenced by genetic introgression from *P. minor*, rather than by a unique evolutionary path. Furthermore, the chloroplast haplotypes of the insular *P. japonica* ecotype almost invariably align with those of *P. minor*, indicating an asymmetrical initial hybridization where *P. minor* primarily acted as the maternal parent in the formation of F1 hybrids.

Asymmetric initial hybridization patterns might be observed when postzygotic reproductive barriers asymmetrically limit the role of *P. japonica* as a female parent in F1 hybrid formation. However, crossing experiments contradict this hypothesis: the viability of F2 hybrids from *P. japonica* (female) × *P. minor* (male) crosses is notably higher compared to those from *P. minor* (female) × *P. japonica* (male) crosses (Takahashi, 2015). We consider that divergent reproductive strategies offer the most plausible explanation for this asymmetry. Outcrossing plants, with their higher insect-mediated pollen exportation ratios, could be asymmetrically transferring pollen to selfers (Pickup et al., 2019). Considering the complete dependence of *P. japonica* on pollinators for fruit set (Ushimaru & Nakata, 2001), along with the predominantly self-pollinating nature of *P. minor* (Suetsugu, 2015b), it is reasonable that *P. japonica* predominantly acts as the pollen donor in the initial hybridization phase. This hybridization pattern, where the selfing species acts as the maternal parent in the initial cross but subsequent gene flow predominantly occurs from the selfing species into the outcrossing species due to backcrossing with the outcrossing species, is common among hybridizing selfing/outcrossing species pairs (Brandvain et al., 2014; Rifkin et al., 2019).

The insular conditions on Kozu Island likely favor introgression from *P. minor* to *P. japonica*, as introgressed *P. japonica* have not been documented in other regions of Japan. Given the limited migration from the mainland, island flora could benefit from introgression, potentially leading to the emergence of novel traits in response to specific biotic or abiotic conditions (Jorgensen & Olesen, 2001; Suetsugu et al., 2024). In extreme cases, such events could even lead to speciation (Hegarty & Hiscock, 2005). Intriguingly, the insular ecotype of *P. japonica* possesses a certain level of private alleles, and its observed heterozygosity is close to the expected heterozygosity, indicating that a considerable number of generations have passed since the ecotype formation. DIYABC analysis suggests that the insular population of *P. japonica* likely originated from an admixture event between *P. japonica* and *P. minor* approximately 4,000 generations ago. Thus, the insular *P. japonica* might be potentially becoming a distinct, endemic lineage. Importantly, considering that the insular *P. japonica* retains most of its genetic components from *P. japonica*, despite pure *P. japonica* being extinct on Kozu Island, backcrossing now likely occurs primarily within the insular ecotype of *P. japonica*, rather than with *P. minor*. Meanwhile, the discovery of hybrid individuals, likely arising from hybridization events between the already introgressed insular *P. japonica* and pure *P. minor*, suggests that hybridization might be an ongoing process on the island.

Our pollination experiments can refute the hypothesis that selection pressure for selfing significantly contributes to the formation of the insular ecotype of *P. japonica* introgressed with *P. minor*. Although the acquisition of selfing as a reproductive assurance mechanism has been well recognized in some endemic plants on the Izu Islands (Inoue, 1993; Inoue & Amano, 1986), a complete dependence on pollinators for fruit set was detected in the insular ecotype of *P. japonica*. Although hybrid individuals have autogamous selfing ability, the insular ecotype of *P. japonica* is more common and widespread than hybrids on Kozu Island. Therefore, we consider the limited role of selfing ability in influencing introgression. Nonetheless, reproductive biology may still play a role in facilitating introgression. Considering the limited pollinator fauna on oceanic islands (Hiraiwa & Ushimaru, 2017; Olesen & Valido, 2003), shared island pollinators, which often have broader ecological roles, might contribute to hybridization events (e.g., Suetsugu et al., 2023). Additionally, the slightly reduced corolla size of insular *P. japonica* could enhance fitness on the island, due to the prevalence of smaller insects on oceanic islands (Barrett, 1996). These possibilities warrant further investigation.

We hypothesize that the unique soil and climate conditions on Kozu Island represent one of the most plausible mechanisms for increased introgression with *P. minor*. Intriguingly, all sites where the insular ecotype of *P. japonica* is found are covered with volcanic rocks formed post-Holocene (less than 11,700 years ago). Given that a generation of *Pogonia* spans only a few years (Takahashi, 2015), this suggests a potential alignment between these geological histories and the admixture event between *P. japonica* and *P. minor* approximately 4,000 generations ago. The volcanic rocks are characterized by well-draining traits (Tsuya, 1930), thereby making them extremely prone to drying out. This likely facilitates the introgression of *P. minor*, which is adapted to arid environments, into the insular ecotype of *P. japonica.* Although insular *P. japonica* can inhabit dry habitats, it may not possess the same drought tolerance as *P. minor*. Kozu Island, compared to other habitats of *Pogonia*, tends to receive a higher amount of precipitation (Supplementary Table S3), which may allow insular *P. japonica* to survive. The combination of abundant rainfall and well-draining volcanic rocks may have facilitated the persistence of the introgressants: insular *P. japonica* may be advantageous in fluctuating environments—moist immediately after rainfall but typically very dry—due to the plasticity resulting from the combination of *P. japonica* and *P. minor* genomes (e.g., Akiyama et al., 2021; Sun et al., 2020).

In summary, our study strongly suggests that introgression from *P. minor*, a species adapted to arid conditions, into the insular ecotype of *P. japonica* has successfully expanded into similar arid environments beyond its typical range. However, further study is needed to fully understand the dynamics of adaptive introgression in *P. japonica.* For instance, a reciprocal translocation experiment might be helpful to clarify the extent to which introgression has enhanced the survival capabilities of the insular ecotype under arid conditions. Additionally, monitoring moisture levels at a fine scale in the natural habitats (e.g., Akiyama et al., 2021) will allow us to explore if the proportion of *P. minor* genes present drives fine-scale niche separation among insular *P. japonica* individuals. Lastly, transcriptomic analysis on insular *P. japonica* can determine if *P. minor* genes are predominantly expressed in arid environments, while *P. japonica* genes are more expressed under moist conditions. These studies will provide a deeper understanding of how genetic exchange between species enables them to thrive in new and changing environments.

## Supplementary material

Supplementary material is available online at *Evolution Letters*.

qrae034_suppl_Supplementary_Figures

qrae034_suppl_Supplementary_Tables

## Data Availability

The raw reads of MIG-seq and MPM-seq data were submitted to the DDBJ Sequence Read Archive under the following BioProject Accessions: MIG-seq data: PRJDB17977; MPM-seq data: PRJDB17978.
