# Supplementary material for: Insular environment-dependent introgression from an arid-grassland orchid to a wetland orchid on an oceanic island
Source: Evol Lett. 2024 Jul 15;8(6):799–812. doi: 10.1093/evlett/qrae034 (PMC11637555; doi:10.1093/evlett/qrae034)
Supplement: qrae034_suppl_Supplementary_Figures [file qrae034_suppl_supplementary_figures.pdf]

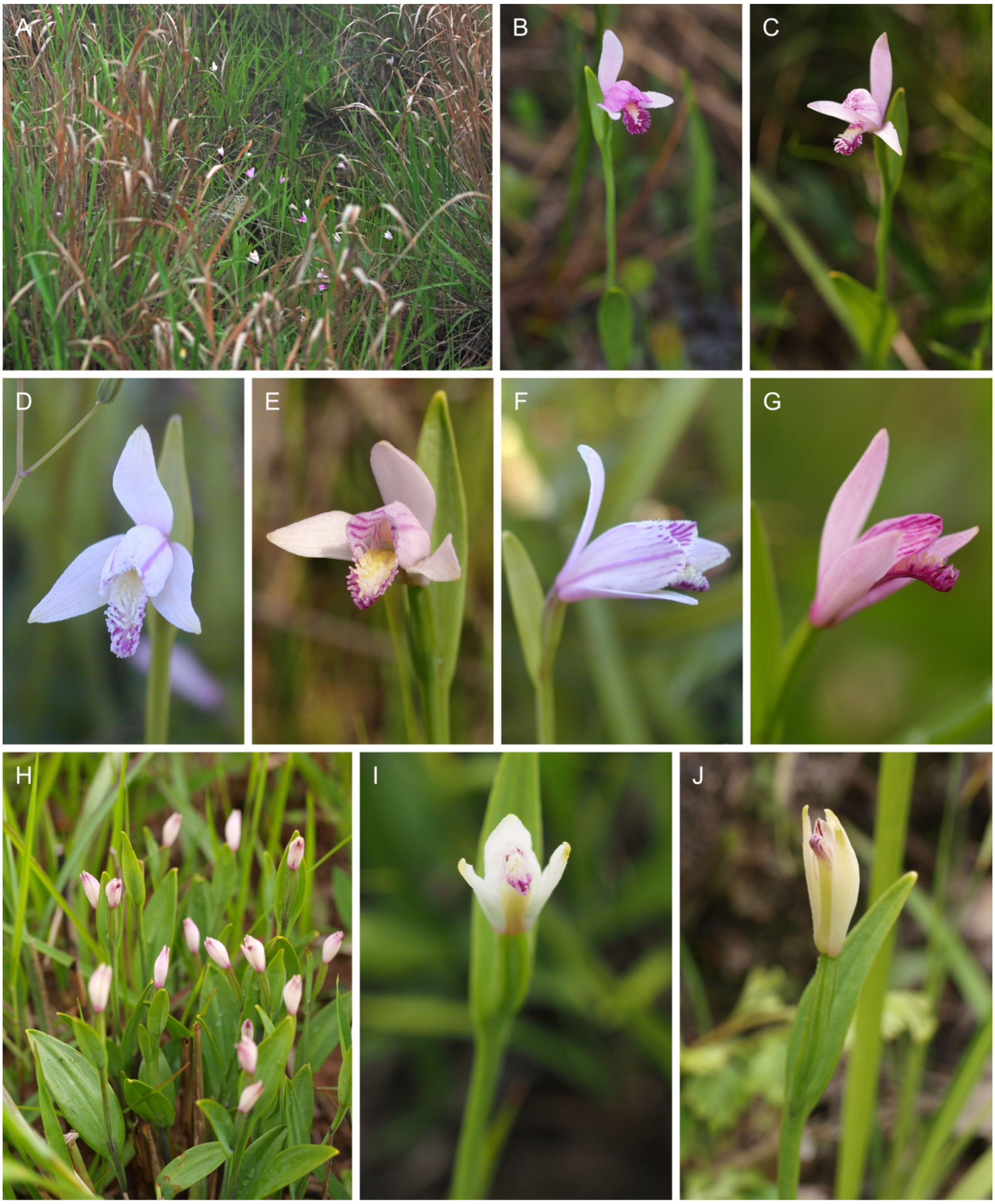

Figure S1. The contrasting habitats of *Pogonia japonica* and *Pogonia minor* on mainland Japan (Fukuoka Pref.). (A) The wetland harboring *P. japonica* flowering plants at Kitakyushu City. (B–G) Flowering plants of *P. japonica* in their wetland habitats. (H) The dry grassland harboring *P. minor* flowering plants at Kanda-cho. (I–J) Flowering plants of *P. minor* in their dry grassland habitats.

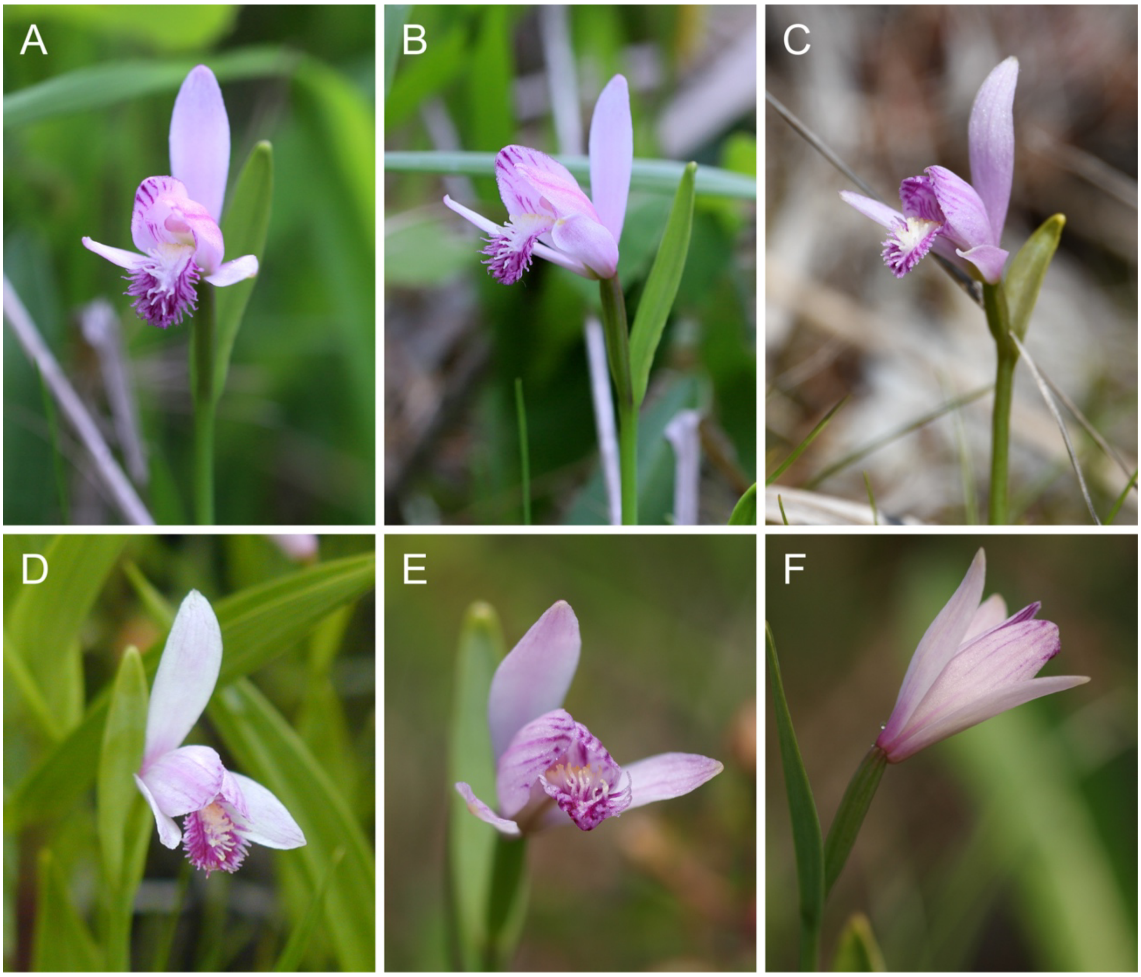

Figure S2. Morphological variation of *Pogonia japonica* flowers on mainland Japan. (A–B). Flowering plants at Ena City, Gifu Pref. (C) Flowering plant at Kato City, Hyogo Pref. (D–F) Flowering plants at Kitakyushu City, Fukuoka Pref.

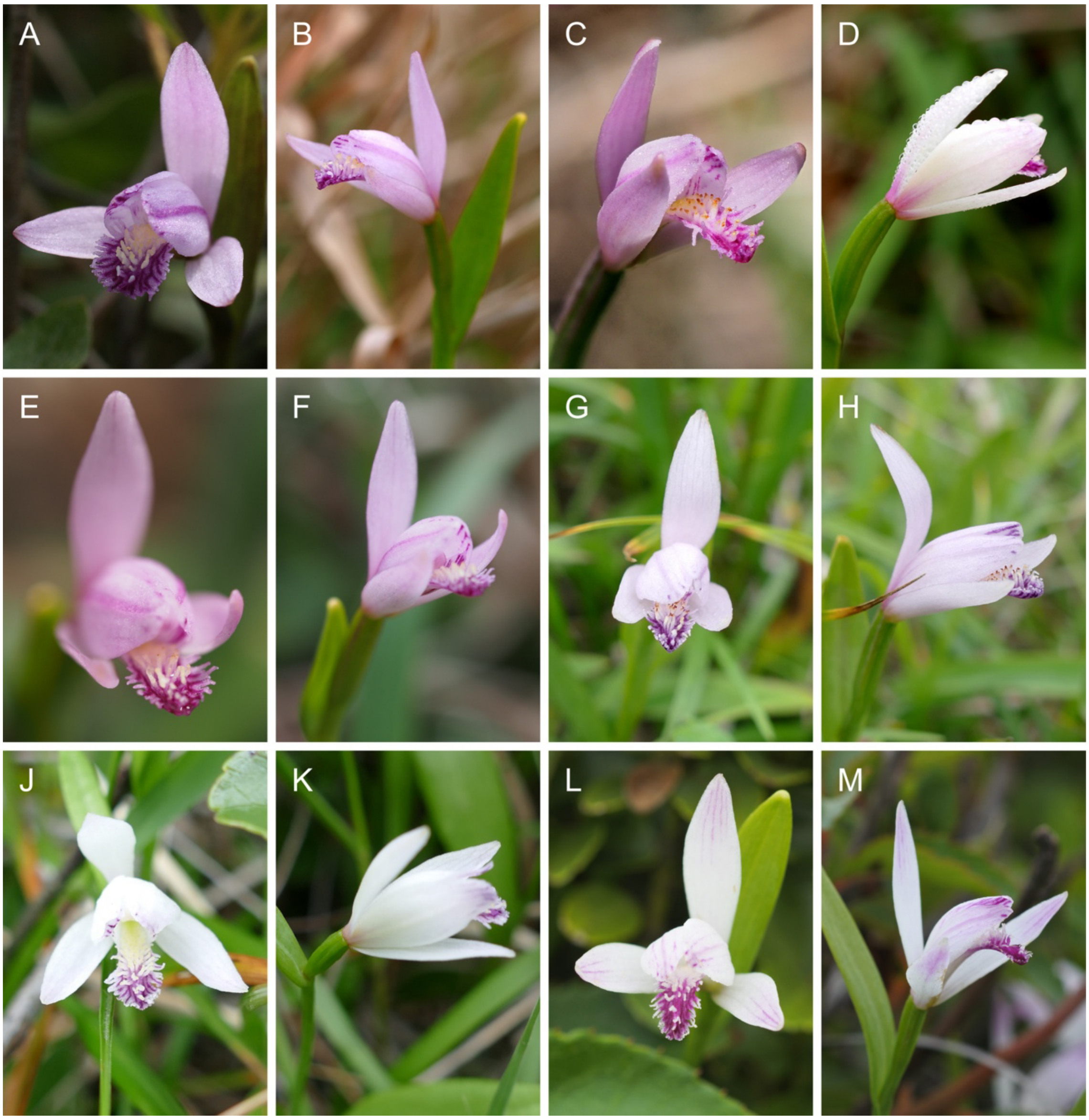

Figure S3. Morphological variation of the insular ecotype of *Pogonia japonica* flowers on Kozu Island.

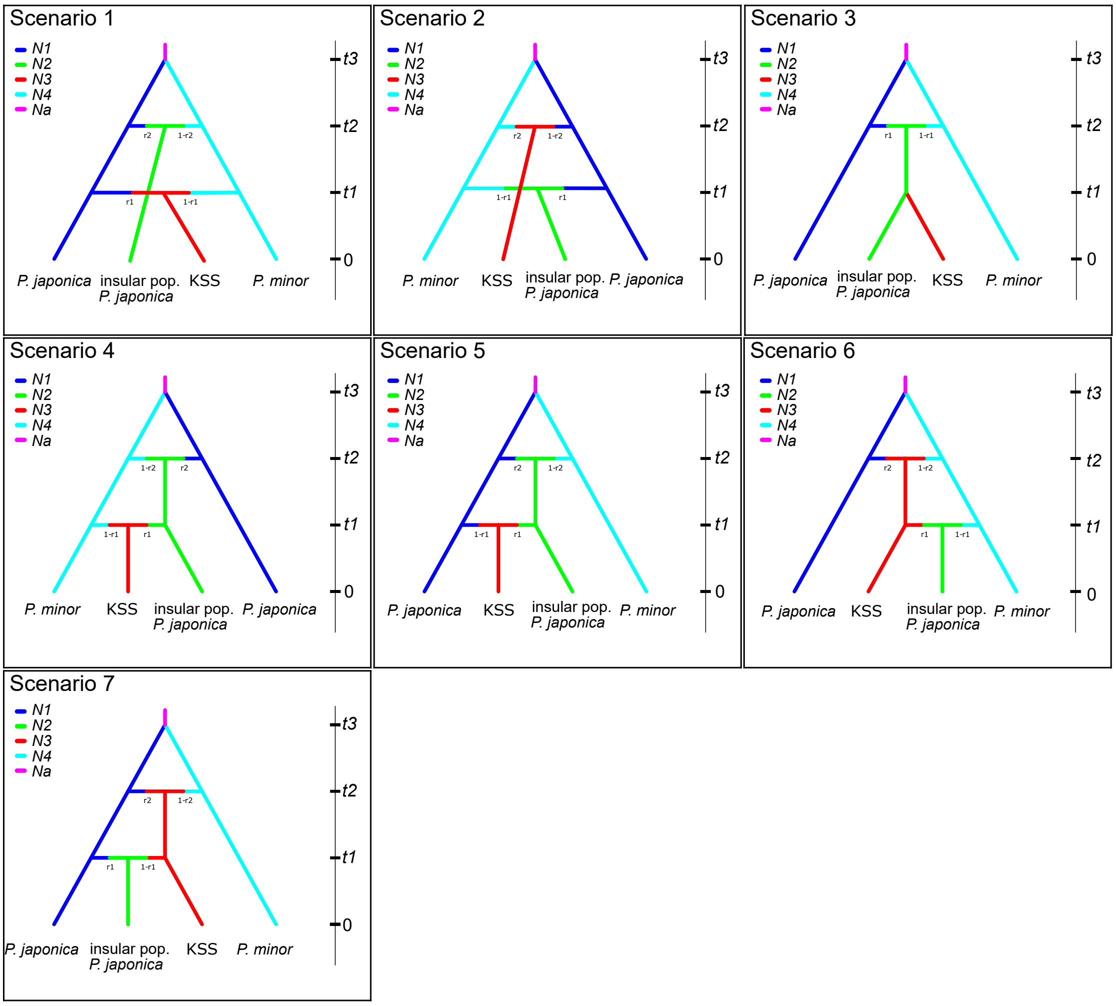

Figure S4. Graphical illustration of admixture scenarios of *Pogonia* in DIYABC analysis. Line colors correspond with population sizes. For descriptions of parameters and their priors, see Table S3.

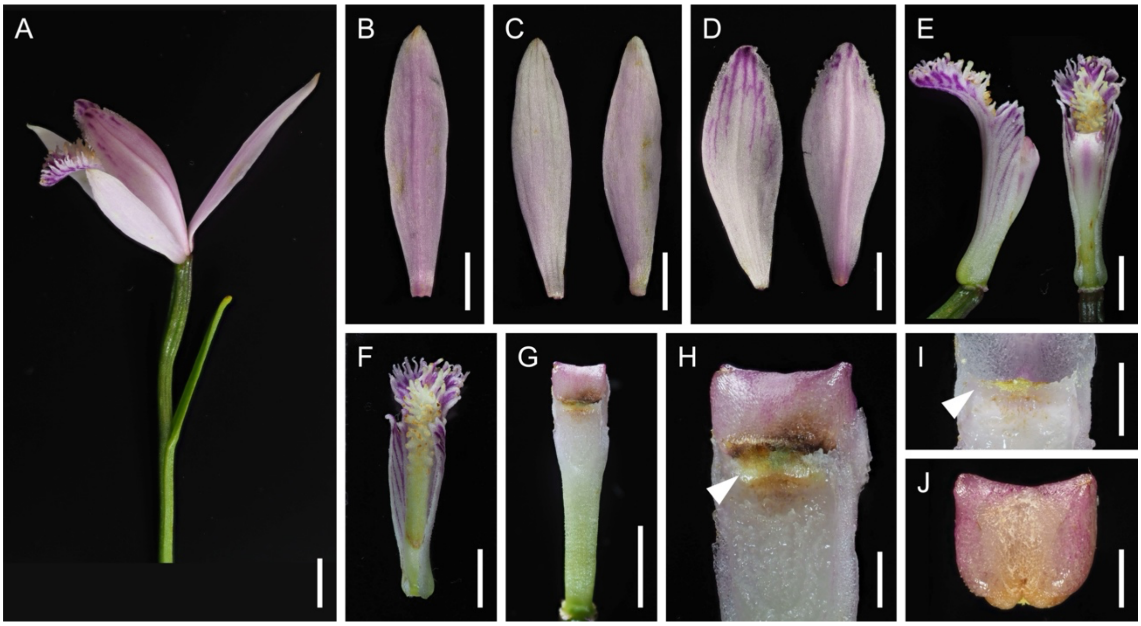

Figure S5. Floral morphology of *Pogonia japonica* on mainland Japan (Ena City, Gifu Pref.). (*Katsumi Iwahori P1*, KYO). (A) Flower with a floral bract. (B) Dorsal sepal (abaxial view). (C) Lateral sepal (left: adaxial view, right: abaxial view). (D) Petal (left: adaxial view, right: abaxial view). (E) Column and labellum (left: lateral view, right: front view). (F) Labellum. (G) Column. (H) Apical part of column. (I) Apical part of column showing functional rostellum. (J) Anther cap. Arrows indicate functional rostellum. Scale bars: 5 mm (A–G) and 1 mm (H–J).

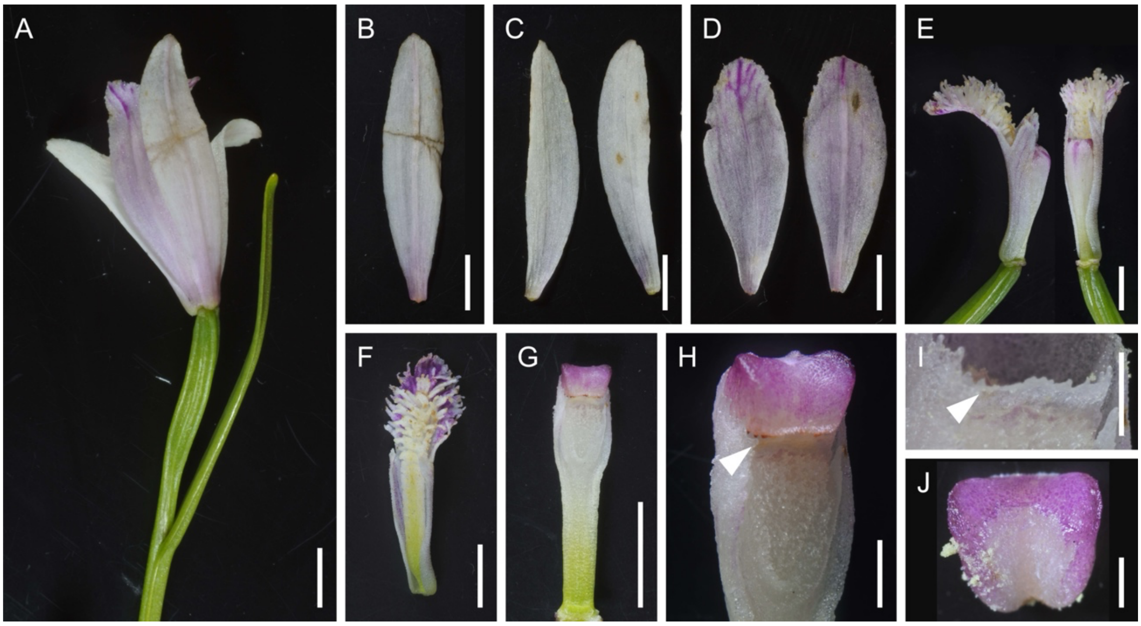

Figure S6. Floral morphology of *Pogonia japonica* on mainland Japan (Kanda-cho, Fukuoka Pref.). (*Koji Tanaka P27*, KYO). (A) Flower with a floral bract. (B) Dorsal sepal (abaxial view). (C) Lateral sepal (left: adaxial view, right: abaxial view). (D) Petal (left: adaxial view, right: abaxial view). (E) Column and labellum (left: lateral view, right: front view). (F) Labellum. (G) Column. (H) Apical part of column. (I) Apical part of column showing functional rostellum. (J) Anther cap. Arrows indicate functional rostellum. Scale bars: 5 mm (A–G) and 1 mm (H–J).

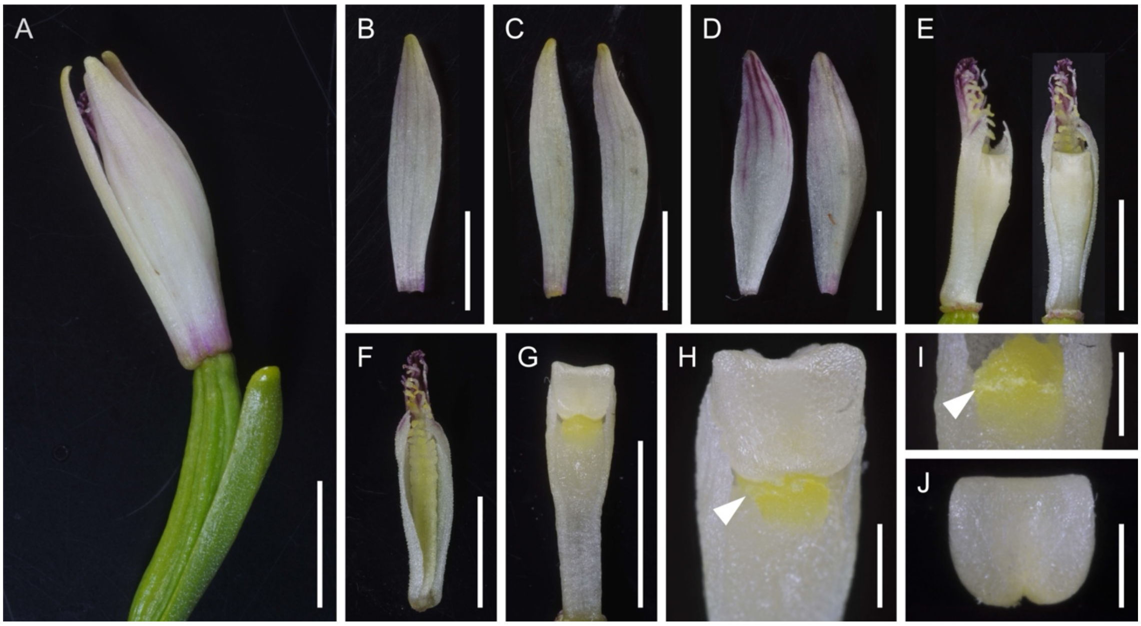

Figure S7. Floral morphology of *Pogonia minor* on mainland Japan (Kitakyushu City, Fukuoka Pref.). (*Koji Tanaka P30*, KYO). (A) Flower with a floral bract. (B) Dorsal sepal (abaxial view). (C) Lateral sepal (left: adaxial view, right: abaxial view). (D) Petal (left: adaxial view, right: abaxial view). I Column and labellum (left: lateral view, right: front view). (F) Labellum. (G) Column. (H) Apical part of column. (I) Apical part of column showing the absence of functional rostellum. (J) Anther cap. Arrows indicate the absence of functional rostellum. Scale bars: 5 mm (A–G) and 1 mm (H–J).

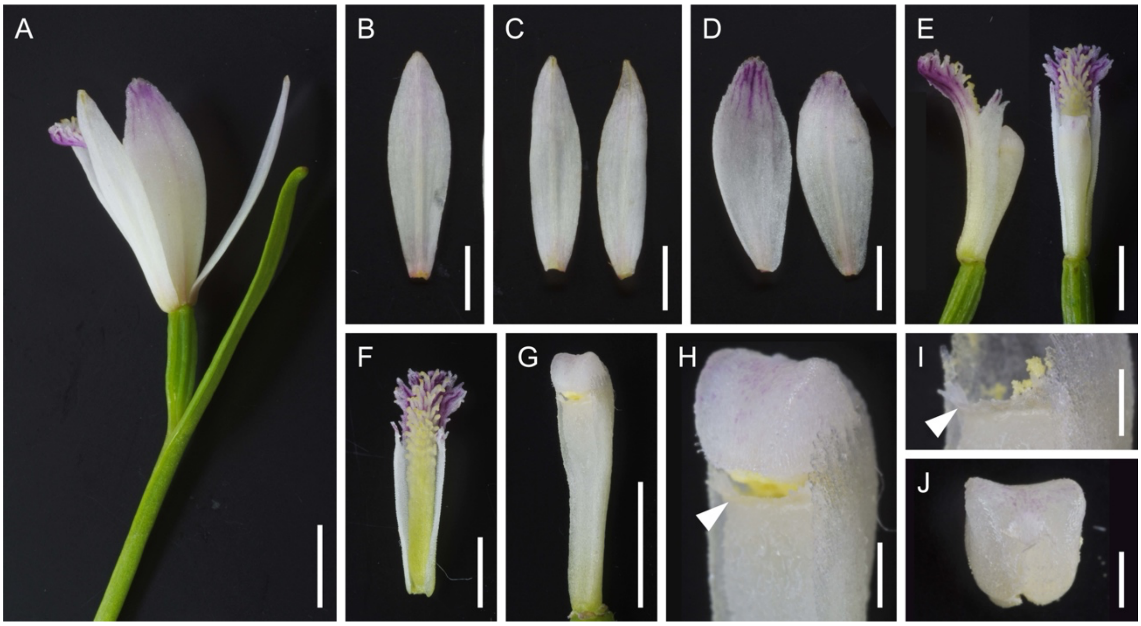

Figure S8. Floral morphology of the insular ecotype of *Pogonia japonica* in Kuroshima, Kozu Island (*Kenji Suetsugu P4*, KYO). (A) Flower with a floral bract. (B) Dorsal sepal (abaxial view). (C) Lateral sepal (left: adaxial view, right: abaxial view). (D) Petal (left: adaxial view, right: abaxial view). (E) Column and labellum (left: lateral view, right: front view). (F) Labellum. (G) Column. (H) Apical part of column. (I) Apical part of column showing semi-degenerated rostellum. (J) Anther cap. Arrows indicate semi-degenerated rostellum. Scale bars: 5 mm (A–G) and 1 mm (H–J).

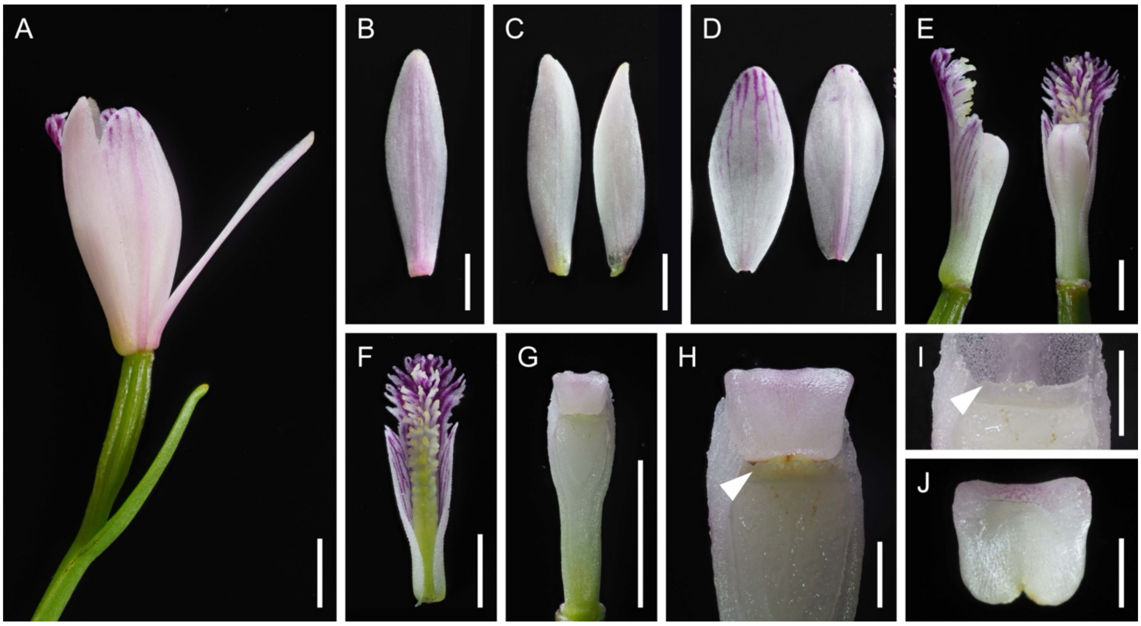

Figure S9. Floral morphology of the insular ecotype of *Pogonia japonica* around Sendai-ike, Kozu Island (*Masayuki Ishibashi P39*, KYO). (A) Flower with a floral bract. (B) Dorsal sepal (abaxial view). (C) Lateral sepal (left: adaxial view, right: abaxial view). (D) Petal (left: adaxial view, right: abaxial view). (E) Column and labellum (left: lateral view, right: front view). (F) Labellum. (G) Column. (H) Apical part of column. (I) Apical part of column showing functional rostellum. (J) Anther cap. Arrows indicates functional rostellum. Scale bars: 5 mm (A–G) and 1 mm (H–J).

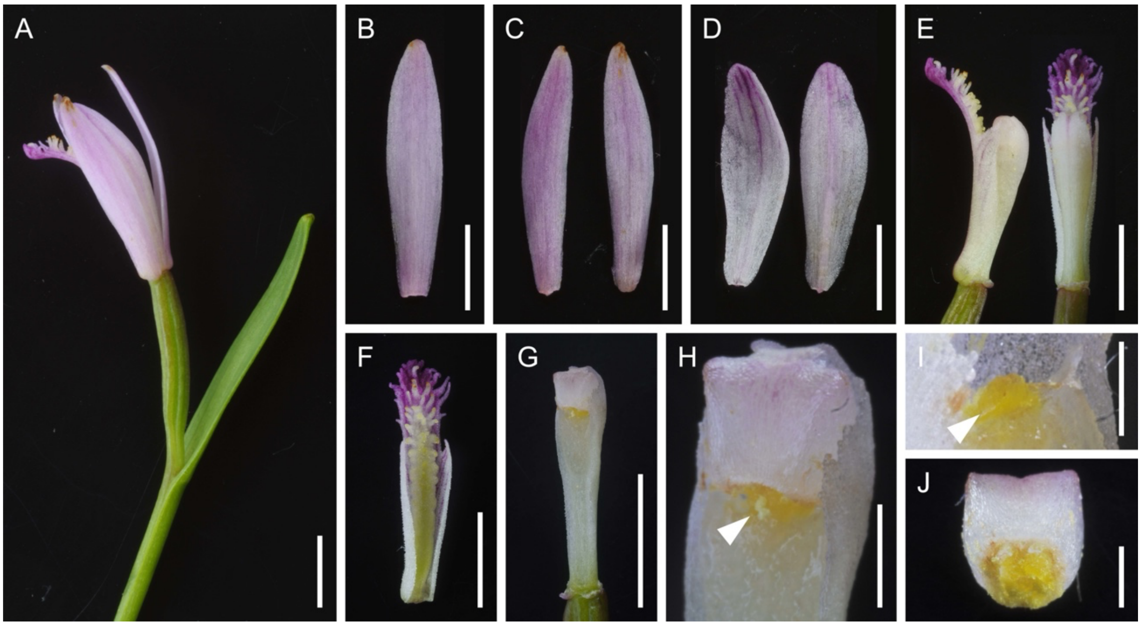

Figure S10. Floral morphology of *Pogonia japonica* × *Pogonia minor* in Kuroshima-shita, Kozu Island (*Kenji Suetsugu P10*, KYO). (A) Flower with a floral bract. (B) Dorsal sepal (abaxial view). (C) Lateral sepal (left: adaxial view, right: abaxial view). (D) Petal (left: adaxial view, right: abaxial view). (E) Column and labellum (left: lateral view, right: front view). (F) Labellum. (G) Column. (H) Apical part of column. (I) Apical part of column showing the absence of functional rostellum. (J) Anther cap. Arrows indicate the contact between pollinia and stigma. Scale bars: 5 mm (A–G) and 1 mm (H–J).

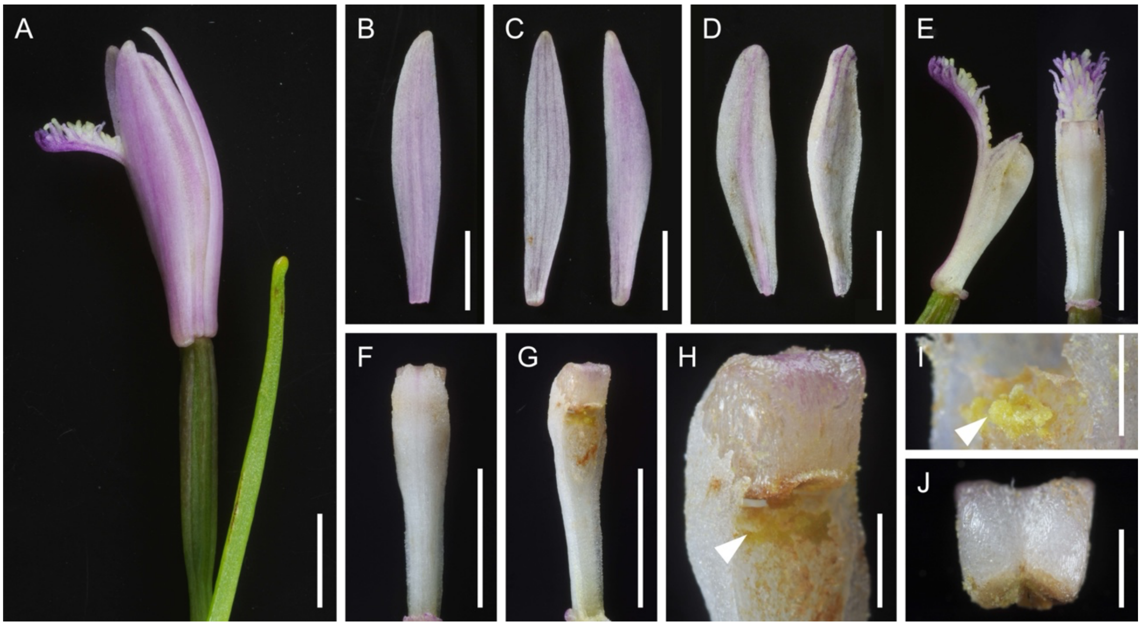

Figure S11. Floral morphology of *Pogonia japonica* × *Pogonia minor* in Kuroshima-shita, Kozu Island (*Kenji Suetsugu P13-2*, KYO). (A) Flower with a floral bract. (B) Dorsal sepal (abaxial view). (C) Lateral sepal (left: adaxial view, right: abaxial view). (D) Petal (left: adaxial view, right: abaxial view). (E) Column and labellum (left: lateral view, right: front view). (F) Column, top view. (G) Column, bottom view. (H) Apical part of column. (I) Apical part of column showing the absence of functional rostellum. (J) Anther cap. Arrows indicate the contact between pollinia and stigma. Scale bars: 5 mm (A–G) and 1 mm (H–J).

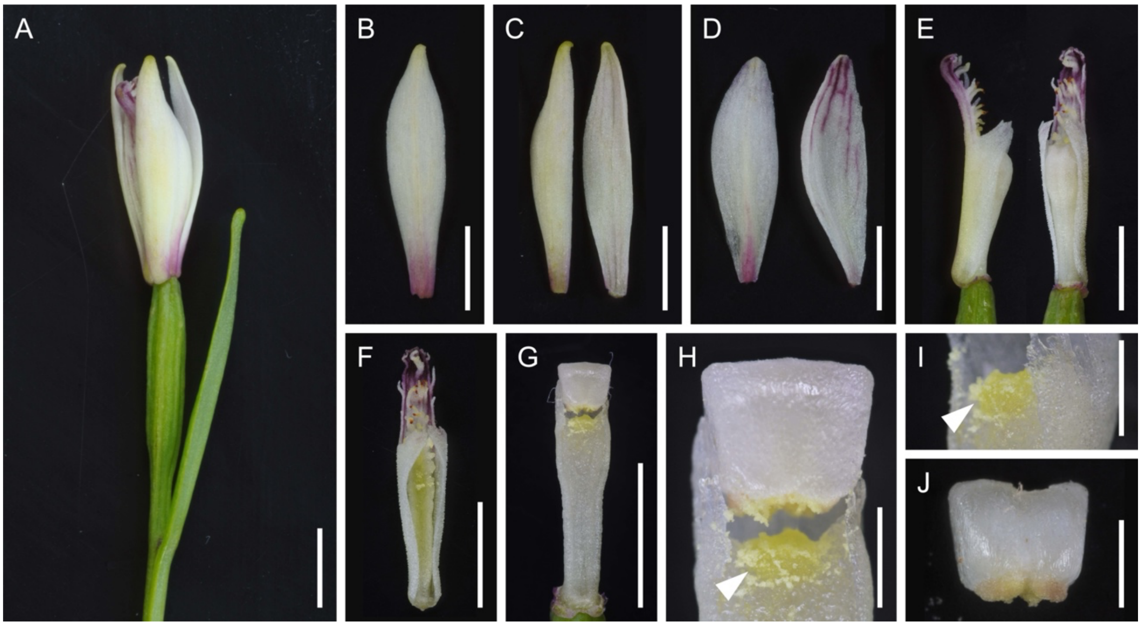

Figure S12. Floral morphology of *Pogonia minor* on Mt. Tenjo, Kozu Island (*Kenji Suetsugu P23*, KYO). (A) Flower with a floral bract. (B) Dorsal sepal (abaxial view). (C) Lateral sepal (left: adaxial view, right: abaxial view). (D) Petal (left: adaxial view, right: abaxial view). (E) Column and labellum (left: lateral view, right: front view). (F) Labellum. (G) Column. (H) Apical part of column. (I) Apical part of column showing the absence of functional rostellum. (J) Anther cap. Arrows indicate the contact between pollinia and stigma. Scale bars: 5 mm (A–G) and 1 mm (H–J).

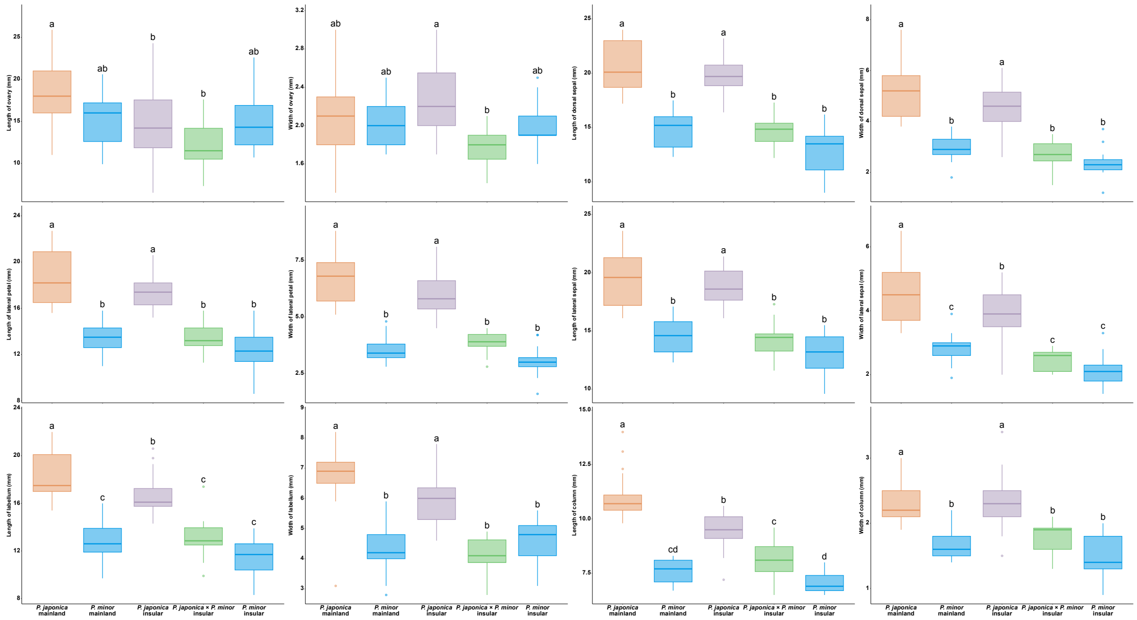

Figure S13. Box-and-whisker plots representing the morphological characteristics of mainland *P. japonica*, the insular ecotype of *P. japonica*, *P. japonica* × *P. minor*, mainland *P. minor*, and insular *P. minor*. The median, quartiles, non-outlier ranges, and outliers are depicted by bold lines, boxes, whiskers, and points, respectively. Letters on the top of each upper whisker indicate the results of the Tukey-Kramer test for each character (P < 0.05).

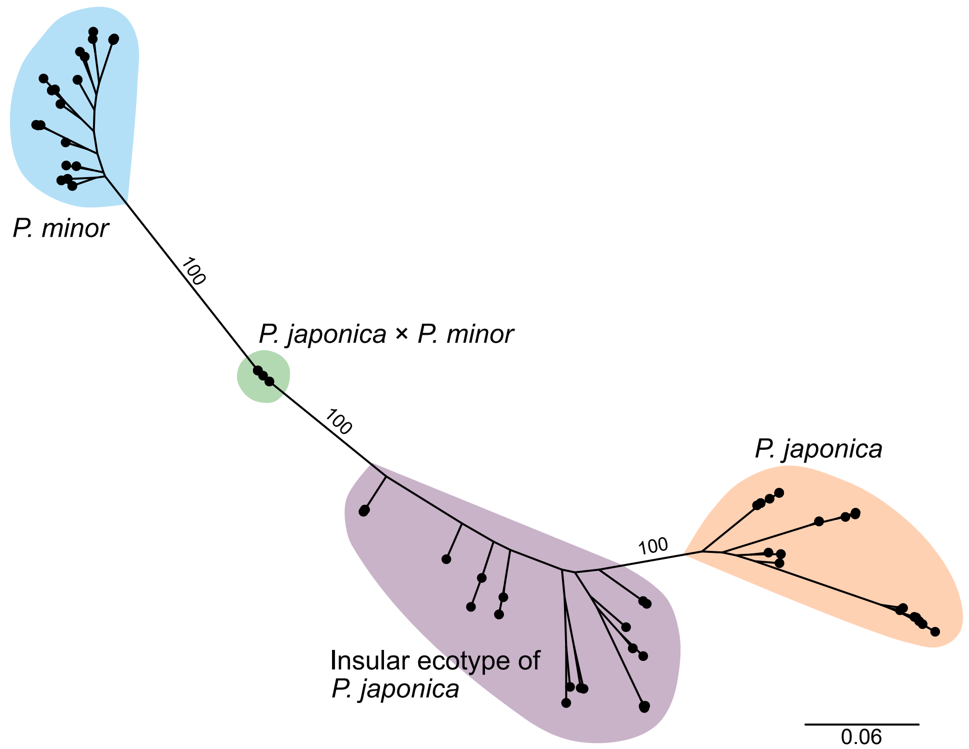

Figure S14. Maximum likelihood phylogenetic tree of mainland *P. japonica*, the insular ecotype of *P. japonica*, *P. japonica* × *P. minor*, and mainland and insular *P. minor* reconstructed using MIG-seq data. Nodes supported by bootstrap values < 70% are not shown. Branch length represents the average number of substitutions per site.

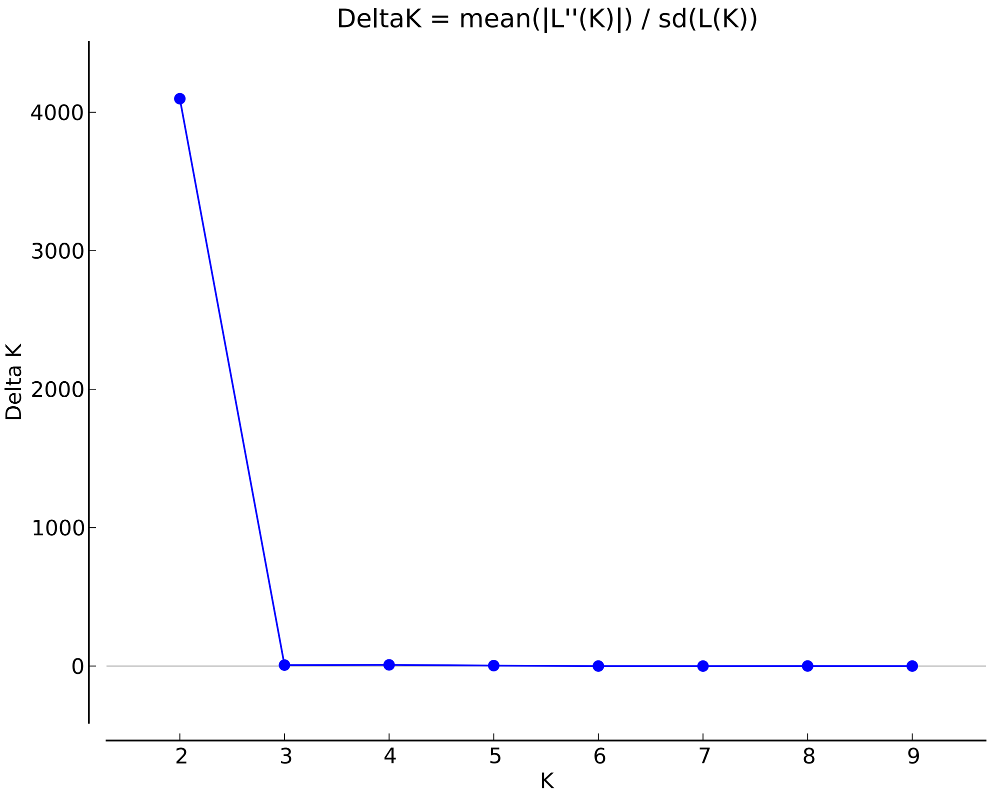

Figure S15. Delta *K* values for each *K* from 1 to 10 calculated using the method of Evanno *et al.* (2005). The largest value was obtained at *K* = 2.
